# Phosphorylation status determines the opposing functions of Smad2/Smad3 as STAT3 cofactors in T_H_17 differentiation

**DOI:** 10.1038/ncomms8600

**Published:** 2015-07-21

**Authors:** Jeong-Hwan Yoon, Katsuko Sudo, Masahiko Kuroda, Mitsuyasu Kato, In-Kyu Lee, Jin Soo Han, Susumu Nakae, Takeshi Imamura, Juryun Kim, Ji Hyeon Ju, Dae-Kee Kim, Koichi Matsuzaki, Michael Weinstein, Isao Matsumoto, Takayuki Sumida, Mizuko Mamura

**Affiliations:** 1 Department of Experimental Pathology, Graduate School of Comprehensive Human Sciences and Faculty of Medicine, University of Tsukuba, 1-1-1 Tennodai, Tsukuba, Ibaraki 305-8575, Japan; 2 Department of Molecular Pathology, Tokyo Medical University, 6-1-1 Shinjuku, Shinjuku-ku, Tokyo 160-8402, Japan; 3 Department of Internal Medicine, Kyungpook National University School of Medicine, 50 SAMDUK-2GA, Jungu, Daegu 700-721, Republic of Korea; 4 Animal Research Center, Tokyo Medical University, 6-1-1 Shinjuku, Shinjuku-ku, Tokyo 160-8402, Japan; 5 Department of Laboratory Animal Medicine, Institute for the 3Rs, College of Veterinary Medicine, Konkuk University, 120 Neungdong-ro, Gwangjin-gu, Seoul 143-701, Republic of Korea; 6 Laboratory of Systems Biology, Center for Experimental Medicine and Systems Biology, The Institute of Medical Science, University of Tokyo, 4-6-1 Shirokanedai, Minato, Tokyo 108-8639, Japan; 7 Department of Molecular Medicine for Pathogenesis, Graduate School of Medicine, Ehime University, Shitsukawa, Toon, Ehime 791-0295, Japan; 8 Department of Rheumatology, Catholic University of Korea, #505, Banpo-Dong, Seocho-Gu, Seoul 137-701, Republic of Korea; 9 College of Pharmacy, Ewha Womans University, 11-1 Daehyun-dong, Seodaemun-gu, Seoul 120-750, Republic of Korea; 10 Department of Gastroenterology and Hepatology, Kansai Medical University, 10-15 Fumizonocho, Moriguchi, Osaka 570-8506, Japan; 11 Department of Molecular Genetics, Ohio State University, 484 West 12th Avenue, Columbus, Ohio 43210, USA; 12 Department of Internal Medicine, University of Tsukuba, 1-1-1 Tennodai, Tsukuba, Ibaraki 305-8575, Japan

## Abstract

Transforming growth factor-β (TGF-β) and interleukin-6 (IL-6) are the pivotal cytokines to induce IL-17-producing CD4^+^ T helper cells (T_H_17); yet their signalling network remains largely unknown. Here we show that the highly homologous TGF-β receptor-regulated Smads (R-Smads): Smad2 and Smad3 oppositely modify STAT3-induced transcription of IL-17A and retinoic acid receptor-related orphan nuclear receptor, RORγt encoded by *Rorc*, by acting as a co-activator and co-repressor of STAT3, respectively. Smad2 linker phosphorylated by extracellular signal-regulated kinase (ERK) at the serine 255 residue interacts with STAT3 and p300 to transactivate, whereas carboxy-terminal unphosphorylated Smad3 interacts with STAT3 and protein inhibitor of activated STAT3 (PIAS3) to repress the *Rorc* and *Il17a* genes. Our work uncovers carboxy-terminal phosphorylation-independent noncanonical R-Smad–STAT3 signalling network in T_H_17 differentiation.

## Abstract (web summary)

TGF-ß and IL-6 are the essential cytokines for mediating the differentiation of IL-17-producing CD4+ T helper cells (T_H_17). Here, Yoon *et al*. provide more insights into this process and describe the opposing roles of TGFß-signalling intermediates Smad2 and Smad3 as STAT3 cofactors in Th17 differentiation.

## Graphical abstract

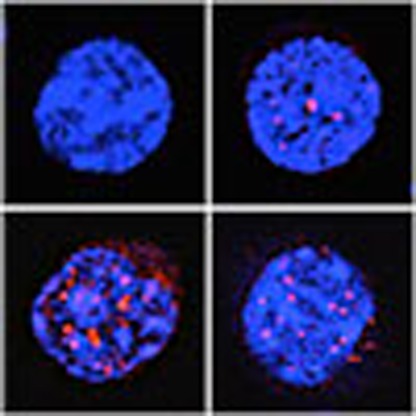

Transforming growth factor-β (TGF-β) had been appreciated as the most potent immunosuppressive cytokine, suppressing the differentiation and functions of effector immune cells as inducing suppressor immune cells^1^,^2^. However, since identified as the requisite cytokine in combination with interleukin (IL)-6 for the differentiation of IL-17-producing CD4^+^ T helper cell (T_H_17) through inducing a master transcription factor, retinoic acid-related orphan receptor-γt (RORγt) and IL-17 (refs 3, 4), context-dependent multidirectional roles of TGF-β have been highlighted in immune regulation, similarly to its roles in carcinogenesis and cancer progression^5^. T_H_17 is a crucial effector CD4^+^ T-cell subset in inflammation, protective mechanisms against infections, tumour immunity and autoimmune responses^6^,^7^. Crucial pathogenic role of T_H_17 in autoimmune diseases such as rheumatoid arthritis (RA) has been well demonstrated by numerous studies including the pioneer work showing the attenuation of collagen-induced arthritis (CIA) in the mice deficient in IL-17A (ref. 8).

Intracellular signal transduction of TGF-β superfamily cytokines is initiated by two types of serine/threonine kinase transmembrane receptors^9^,^10^. TGF-β ligands bind to TGF-β type II receptor (TβRII), which transphosphorylates and activates TGF-β type I receptor (TβRI). TGF-β-specific receptor-regulated Smads (R-Smads), Smad2 and Smad3, are composed of N-terminal Mad homology-1 (MH1) domain, linker region and carboxy-terminal (C-terminal) MH2 domain that contains two serine residues phosphorylated by TβRI. Activated TβRI phosphorylates serine residues, SSXS in MH2 domains of R-Smads, which form the heterodimer complex with common-mediator Smad, Smad4, to regulate transcription of the target genes^10^,^11^. TβRI phosphorylates not only the C-termini of R-Smads but also activates various protein kinases including mitogen-activated protein kinases (MAPKs): extracellular signal-regulated kinase (ERK), c-Jun N-terminal kinase (JNK) and p38 MAPK (p38), which then phosphorylate the variable linker regions of R-Smads^11^,^12^,^13^. MAPKs are shared by T-cell receptor (TCR) and various cytokines including IL-6 as the crucial intracellular signalling mediators for effector T-cell differentiation^14^,^15^.

IL-6 and other cytokines such as IL-21 and IL-23 that induce and maintain T_H_17 activate STAT3, a critical transcription factor for T_H_17 differentiation and the pathogenesis of autoimmune diseases including RA^16^. In contrast to the established essential roles of STAT3-mediated IL-6 signalling in T_H_17 differentiation, molecular mechanisms by which R-Smads regulate T_H_17 differentiation still remain under debate. Despite their high amino-acid sequence homology, Smad2 and Smad3 exert both redundant and distinct functions in TGF-β signalling depending on the context^17^. Several reports have shown contradictory results regarding their roles in T_H_17 differentiation: Smad2 as the essential inducer^18^,^19^, Smad3 as the negative regulator^20^,^21^ or non-Smad signals as the crucial inducers with irrelevance of R-Smads^22^,^23^. Thus far, roles of canonical TGF-β signalling through C-terminal phosphorylation of R-Smads for T_H_17 differentiation have been examined. MAPK signalling pathways, which phosphorylate linker regions of R-Smads, play crucial roles in differentiation and functions of effector T cells^1^,^2^,^24^. However, whether diverse phosphorylation status of R-Smads, such as linker phosphorylation or unphosphorylation, affects T_H_17 differentiation remains largely undetermined.

In this study, we seek to determine the mechanisms whereby R-Smads regulate T_H_17 differentiation. We investigate the molecular mechanisms how Smad2 and Smad3 regulate the transcription of the essential genes for T_H_17 and examine the pathophysiological roles of R-Smads in T_H_17-related inflammatory disease by applying a CIA model to Smad2-deficient (*Smad2*^−/−^), Smad3-deficient (*Smad3*^−/−^) and control wild-type mice. We discover the opposing functions of Smad2 and Smad3 as transcription cofactors of STAT3 in T_H_17 differentiation independently of Smad4: the canonical partner of C-terminally phosphorylated R-Smads. Mechanistic studies show that the phosphorylation status of R-Smads distinctively modulates STAT3-induced transcription of the *Rorc* and *Il17a* genes. Linker-phosphorylated Smad2 (pSmad2L) at the residue Ser255 via ERK serves as a STAT3 co-activator in cooperation with p300, whereas C-terminally unphosphorylated Smad3 (unphosphorylated Smad3C) serves as a STAT3 co-repressor in cooperation with protein inhibitor of activated STAT3 (PIAS3), the negative regulator of STAT3 signalling.

## Results

### Opposing roles of Smad2 and Smad3 in CIA

**Figure 1:**
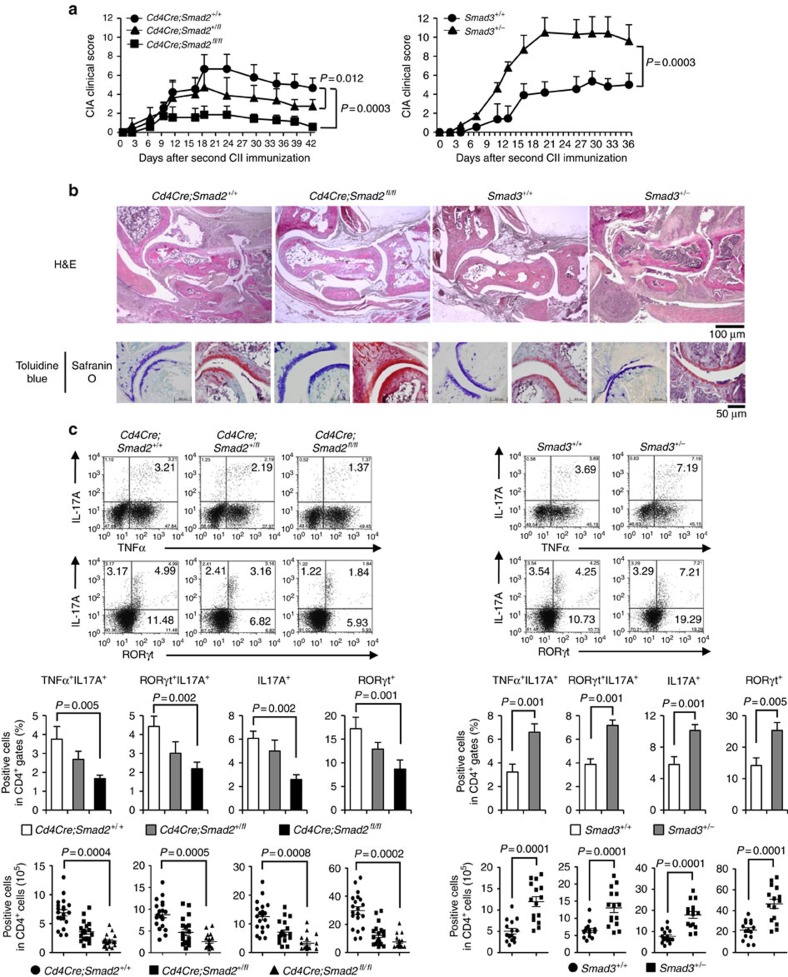
Opposing effects of Smad2 and Smad3 on T_H_17 response in CIA. *Cd4Cre;Smad2*^*+/+, +/fl, fl/fl*^ and *Smad3*^*+/+, +/−*^ mice were immunized with type II collagen emulsified in complete Freund adjuvant twice in 3 weeks interval to induce CIA. (**a**) CIA scoring courses of *Cd4Cre;Smad2*^*+/+*^, *Cd4Cre;Smad2*^*+/fl*^, *Cd4Cre;Smad2*^*fl/fl*^ mice (left, *n*=11/*Cd4Cre;Smad2* genotype) and *Smad3*^*+/+*^, *Smad3*^*+/−*^ mice (right, *n*=13/*Smad3* genotype) with *P* values (two-way analysis of variance (ANOVA) test). (**b**) Pathological analyses of the joint sections (haematoxylin and eosin, H&E, magnification, × 40, scale bar, 100 μm, toluidine blue and safranin O, magnification, × 200, scale bar, 50 μm). (**c**) Flow cytometry analyses of IL-17A^+^TNF-α^+^ CD4^+^ T cells and RORγt^+^IL-17A^+^ CD4^+^ T cells in the draining lymph nodes of *Cd4Cre;Smad2*^*+/+,+/fl, fl/fl*^ (*n*=20/*Cd4Cre;Smad2* genotype) and *Smad3*^*+/+,+/−*^ mice (*n*=15/*Smad3* genotype) on day 14 after second immunization. Graphs show the percentages and cell numbers of IL-17A^+^, RORγt^+^, IL-17A^+^TNF-α^+^ and IL-17A^+^RORγt^+^ in CD4^+^ gates in the draining lymph nodes. Data are from one experiment representative of seven (**a**,**b**), four (**c**, *Cd4Cre;Smad2*) or three (**c**, *Smad3*) independent experiments. Graphs show mean+s.d. with *P* values (unpaired Student's *t*-test).

To examine the pathophysiological roles of R-Smads in T_H_17-mediated inflammatory disease, we applied a CIA model to T-cell-specific (*Cd4Cre;Smad2*^*+/+, +/fl, fl/fl*^), inducible systemic (*Mx-1Cre;Smad2*^*+/+, +/fl, fl/fl*^) Smad2 conditional knockout mice and Smad3 heterozygote (*Smad3*^+/+, +/−^) mice. They showed normal immune phenotypes with C57BL/6 background in a specific pathogen-free environment (Supplementary Fig. 1), indicating that R-Smads are dispensable for immune homeostasis. T-cell-specific and systemic deletion of Smad2 ameliorated, whereas Smad3 heterozygosity exacerbated CIA (Fig. 1a and Supplementary Fig. 2a). Because both systemic and T-cell-specific deletion of Smad2 showed the same phenotype (Supplementary Fig. 2a,b), we used *Cd4Cre;Smad2*^*+/+,+/fl. fl/fl*^ mice for further study. *Cd4Cre;Smad2*^*fl/fl*^ mice showed significant amelioration in joint lesions, whereas *Smad3*^*+/−*^ mice showed proliferative detritic synovitis with mononuclear cell infiltration and joint destruction (Fig. 1b, upper). Evaluation of proteoglycan and mucopolysaccharide of cartilage by staining with toluidine blue and Safranin O showed the significant maintenance of cartilages in *Cd4Cre;Smad2*^*fl/fl*^ mice and marked destruction of cartilages in *Smad3*^*+/−*^ mice (Fig. 1b, lower). Accumulation of CD4^+^, RORγt^+^ and IL-17A^+^ cells in the joint lesions was ameliorated in *Cd4Cre;Smad2*^*fl/fl*^ mice, whereas it was exacerbated in *Smad3*^*+/−*^ mice (Supplementary Figs 3–5). Consistent with the joint lesions, IL-17A^+^, RORγt^+^, IL-17A^+^TNF-α^+^ and IL-17A^+^RORγt^+^ CD4^+^ T cells decreased in the draining lymph nodes of the arthritic joints of *Cd4Cre;Smad2*^*fl/fl*^ mice and *Mx-1Cre;Smad2*^*fl/fl*^ mice, whereas they increased significantly in those of *Smad3*^*+/−*^ mice (Fig. 1c and Supplementary Fig. 2a). Smad genotypes did not affect other effector T-cell subsets, such as IL-6^+^CD4^+^, TNF-α^+^CD4^+^, T_H_1 (T-bet^+^IFN-γ^+^CD4^+^), natural and inducible T_reg_ cells (CD103^−^Foxp3^+^CD4^+^, CD103^+^Foxp3^+^CD4^+^), naive and memory CD4^+^ (CD44^low^CD62L^high^, CD44^high^CD62L^low^) and CD8^+^ (CD44^low^, CD44^high^) T cells in the draining lymph nodes of the arthritic joints (Supplementary Fig. 6). Thus, Smad2 and Smad3 have the opposing roles in T_H_17 differentiation in the pathogenesis of CIA.

### Opposing functions of Smad2 and Smad3 as STAT3 cofactors

**Figure 2:**
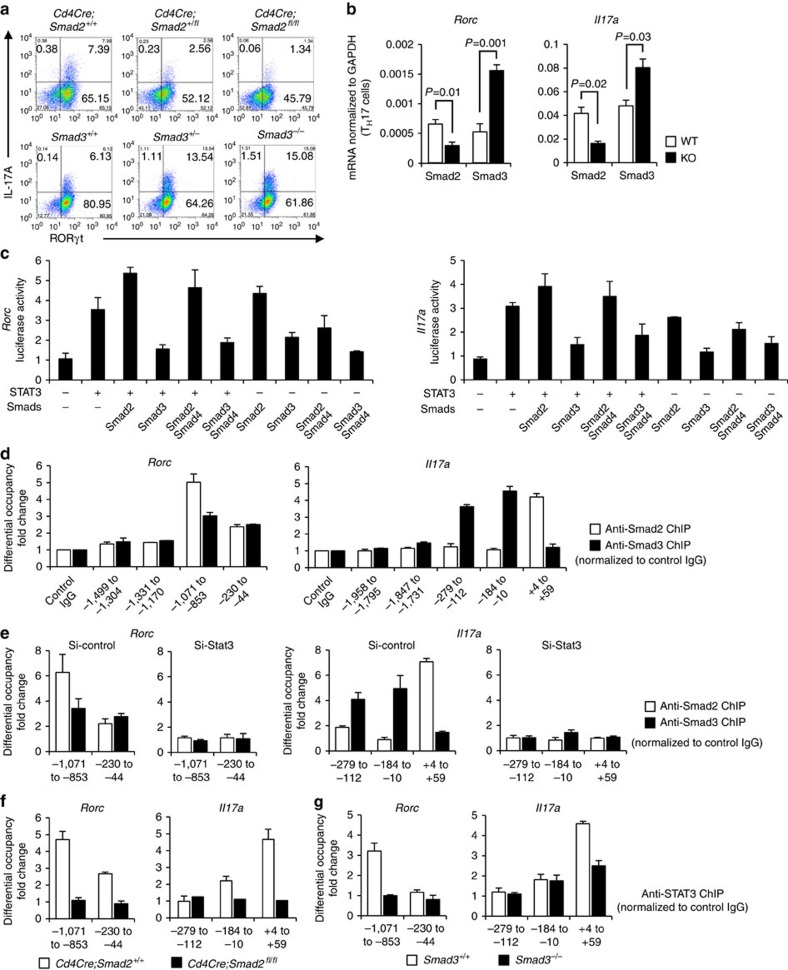
Opposing roles of Smad2 and Smad3 in STAT3-induced T_H_17 differentiation. Purified CD4^+^ T cells were activated under T_H_17-polarizing condition for 3 days. (**a**) Flow cytometry analyses of IL-17A and RORγt in *Smad2*^*+/+,+/−, −/−*^ and *Smad3*^*+/+,+/−, −/−*^ CD4^+^ T cells. (**b**) Quantitative RT–PCR analysis of the *Il17a* and *Rorc* mRNA in *Smad2*^*+/+, −/−*^ and *Smad3*^*+/+, −/−*^ T_H_17 cells (*n*=7). (**c**) Effects of Smads on STAT3-induced activation of the *Rorc* promoter and the *Il17a* promoter constructs transfected in T_H_17 cells were analysed using luciferase assay. (**d**) Binding of Smad2 and Smad3 to the proximal promoter regions of the *Rorc* gene and the *Il17a* gene in T_H_17 cells was determined using ChIP. (**e**) Requirement of STAT3 for the binding of Smad2 and Smad3 to the proximal promoter regions of the *Rorc* gene and the *Il17a* gene was determined with ChIP using STAT3 knockdown T_H_17 cells. Requirement of Smad2 and Smad3 for the binding of STAT3 to the proximal promoter regions of the *Rorc* gene and the *Il17a* gene was determined with ChIP using (**f**) *Smad2*^*−/−*^ or (**g**) *Smad3*^*−/−*^ T_H_17 cells. ChIP data are shown as differential occupancy fold changes. Data are from one experiment representative of seven (**a**,**d**), three (**c**), two (**e**) or five (**f**,**g**) independent experiments or pooled from seven experiments (**b**). Each experiment (**a**–**g**) was performed in triplicate (*n*=3). Data are mean+s.d. or mean+s.d. with *P* values (**b**, unpaired Student's *t*-test).

IL-6 is the main arthritogenic cytokine and TGF-β is produced and activated in the inflammatory lesions^1^,^2^,^25^. Because IL-6 and TGF-β are the pivotal cytokines to induce T_H_17 differentiation, we cultured *Smad2*^−/−^ or *Smad3*^−/−^ CD4^+^ T cells under T_H_17-polarizing condition with IL-6 and TGF-β (ref. 3) to examine the mechanisms whereby R-Smads regulate T_H_17 differentiation. Expression levels of protein and mRNA of RORγt and IL-17A decreased in *Smad2*^−/−^ CD4^+^ T cells, whereas those increased in *Smad3*^−/−^ CD4^+^ T cells (Fig. 2a,b). The mRNA levels of T_H_17-inducing genes (*Batf*, *Il23r*, *Il6*, *Il6ra*, *Il21* and *Il21r*) and T_H_17-suppressing genes (*Il2*, *Il2ra*, *Tbet* and *Eomesodermin*) were unaffected in both *Smad2*^−/−^ and *Smad3*^−/−^ CD4^+^ T cells (Supplementary Fig. 7), suggesting that R-Smads regulate T_H_17 differentiation by specifically targeting the *Rorc* and *Il17a* genes. Because IL-6 or TGF-β alone has little effect on T_H_17 differentiation^3^ and STAT3-mediated IL-6 signalling is crucial for T_H_17 differentiation^16^, we examined whether R-Smads regulate STAT3-induced transcription of RORγt and IL-17A in CD4^+^ T cells cultured under T_H_17-polarizing condition by promoter assays with the luciferase reporters spanning 2 kb upstream of the first exons of the *Rorc* and *Il17a* genes (Fig. 2c). STAT3 or Smad2 alone induced their promoter activities, whereas Smad3 alone had no effect. Smad2 further enhanced, whereas Smad3 suppressed STAT3-induced reporter activation. Co-transfection of Smad4 with R-Smads and STAT3 did not show the additive effects. We next determined the binding of R-Smads to the proximal promoter regions of the *Rorc* and *Il17a* genes in T_H_17 cells by chromatin immunoprecipitation (ChIP) using the primers to detect the DNA-binding sequences of Smads and STAT3 (refs 10, 26, 27). Smad2 and Smad3 were bound to the same sites in the *Rorc* promoter, whereas they were bound to the distinct sites in the *Il17a* promoter (Fig. 2d). Active promoters are characterized by histone acetylation and trimethylation of H3K4, whereas repressed inactive chromatin is marked by methylation of H3K27 and H3K9 (ref. 28). Smad2-binding sites in the *Il17a* promoter showed higher acetylation of histone H3 and trimethylation of histone H3K4, which correlate with transcriptionally active chromatin (Supplementary Fig. 8a). By contrast, Smad3-binding sites in the *Il17a* promoter showed higher trimethylation of histone H3K27, which correlate with transcriptionally inactive chromatin (Supplementary Fig. 8b). These data suggest that Smad2 and Smad3 have the opposing roles in STAT3-induced transcription of the *Rorc* and *Il17a* genes.

We next examined whether STAT3 was necessary for R-Smads to bind to these sites by STAT3 knockdown using short interfering RNA (siRNA) in T_H_17 cells (Supplementary Fig. 9). STAT3 knockdown completely abolished the binding of R-Smads to these sites (Fig. 2e). We then confirmed whether R-Smads are sufficient for STAT3 to bind to these sites using *Smad2*^*−/−*^ and *Smad3*^*−/−*^ T_H_17 cells. STAT3 bound to the Smad2/3-binding sites in the *Rorc* promoter or the Smad2-binding site in the *Il17a* promoter (Fig. 2f,g, white bars). Deficiency of Smad2 or Smad3 prevented STAT3 from binding to these sites (Fig. 2f,g, black bars). Thus, R-Smads and STAT3 are mutually required to bind to the proximal promoters of the *Rorc* and *Il17a* genes. Taken together, Smad2 functions as a transcription co-activator, whereas Smad3 functions as a transcription co-repressor of STAT3 in T_H_17 differentiation.

### Linker-phosphorylated Smad2 induces T_H_17 differentiation

**Figure 3:**
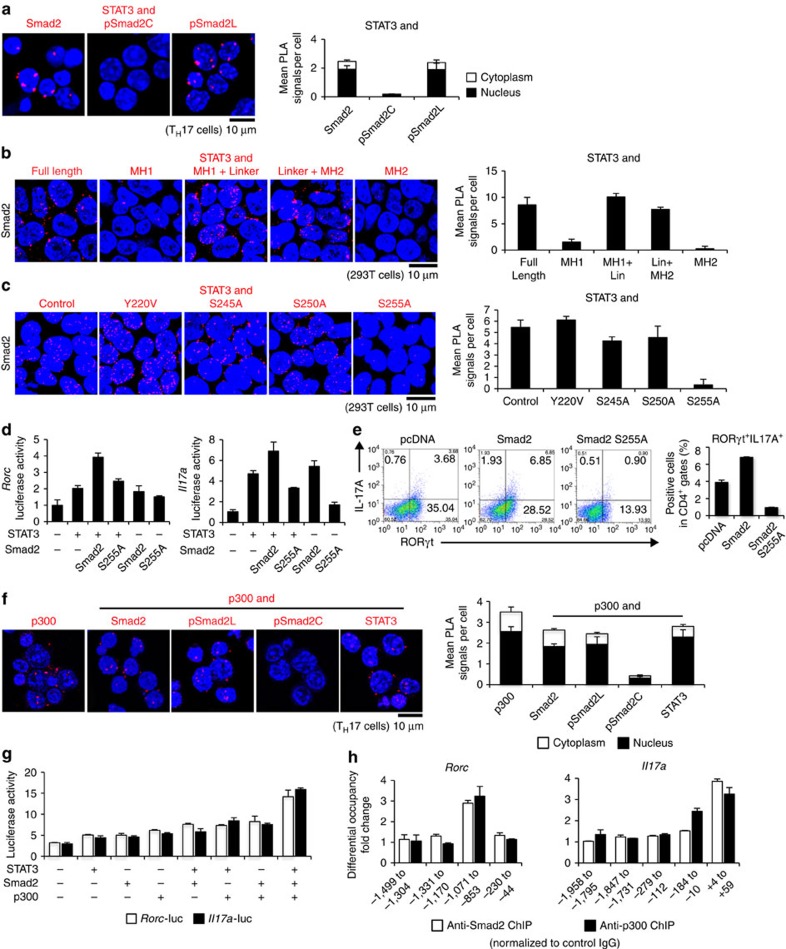
Linker-phosphorylated Smad2 (S255)–STAT3–p300 complex transactivates the *Rorc* and *Il17a*. Interactions of endogenous proteins in T_H_17 cells and exogenous proteins in 293T cells were determined by PLA. PLA signals (**a**–**c**,**f**) were quantified using the BlobFinder software (scale bars, 10 μm; nucleus, black; cytoplasm, white, *n*=10 fields). (**a**) Endogenous interaction between Smad2 and STAT3 in T_H_17 cells. (**b**) Effects of truncated mutations in Smad2 on the interaction with STAT3 in 293T cells. (**c**) Effects of linker domain variations in Smad2 on the interaction with STAT3 in 293T cells. (**d**) Effects of Smad2 (S255A) on STAT3-induced activation of the *Rorc* promoter and the *Il17a* promoter constructs transfected in T_H_17 cells were analysed by luciferase assay. (**e**) Flow cytometry analyses of IL-17A^+^RORγt^+^CD4^+^ T cells transduced with the indicated DNA constructs using Nucleofector (*n*=2). (**f**) Endogenous interactions between p300 and Smad2 or STAT3 in T_H_17 cells were determined using PLA. (**g**) Effects of p300 on Smad2/STAT3-induced activation of the *Rorc* promoter (white) and the *Il17a* promoter (black) constructs transfected in 293T cells were analysed by luciferase assay. (**h**) Binding of Smad2 (white) and p300 (black) to the proximal promoter regions of the *Rorc* gene and the *Il17a* gene in T_H_17 cells was determined using ChIP. ChIP data are shown as differential occupancy fold changes. Data are from one experiment representative of six (**a**), three (**b**–**d**) or two (**e**–**h**) independent experiments. Each experiment (**d**,**g**,**h**) was performed in triplicate (*n*=3). Data are mean+s.d.

We investigated the mechanism how Smad2 functions as a transcription co-activator of STAT3. Proximity ligation assays (PLA) confirmed the endogenous close proximity between Smad2 and STAT3 in T_H_17 cells (Fig. 3a, left). We found that pSmad2L had close proximity with STAT3 in T_H_17 cells (Fig. 3a, right). By contrast, C-terminally phosphorylated Smad2 (pSmad2C) did not show close proximity with STAT3 (Fig. 3a, middle). PLA and immunoprecipitation of 293T cells transfected with the various deletion mutants of Smad2 showed that Smad2 linker deletion mutants (MH1 and MH2)^29^ failed to bind with STAT3 (Fig. 3b and Supplementary Fig. 10a). Transfection of the linker variants of Smad2 showed that the mutant of the linker serine residue 255 to alanine, Smad2 (S255A)^30^, failed to bind with STAT3 (Fig. 3c and Supplementary Fig. 10b). Luciferase reporter assays showed that Smad2 (S255A) failed to enhance STAT3-induced activation of the *Rorc* and *Il17* a promoters (Fig. 3d). Overexpression of Smad2 (S255A) in CD4^+^ T cells cultured under T_H_17-polarizing condition impaired T_H_17 differentiation (Fig. 3e). Therefore, pSmad2L (Ser255) is essential for T_H_17 differentiation.

The histone acetyl-transferase p300 is a crucial transcription co-activator of Smads^9^,^31^. PLA showed that STAT3 and pSmad2L, but not pSmad2C, had the close proximity with p300 in T_H_17 cells (Fig. 3f). Luciferase reporter assays confirmed that p300 further enhanced Smad2/STAT3-induced activation of the *Rorc* and *Il17* a promoters in 293T cells (Fig. 3g). Smad2, STAT3 and p300 bound to the same sites in the proximal promoters of the *Rorc* and *Il17a* genes in T_H_17 cells (Fig. 3h). Thus, pSmad2L (Ser255) forms complex with p300 and STAT3 to bind to the proximal promoter of the *Rorc* and *Il17a* genes.

### Unphosphorylated Smad3 suppresses T_H_17 differentiation

**Figure 4:**
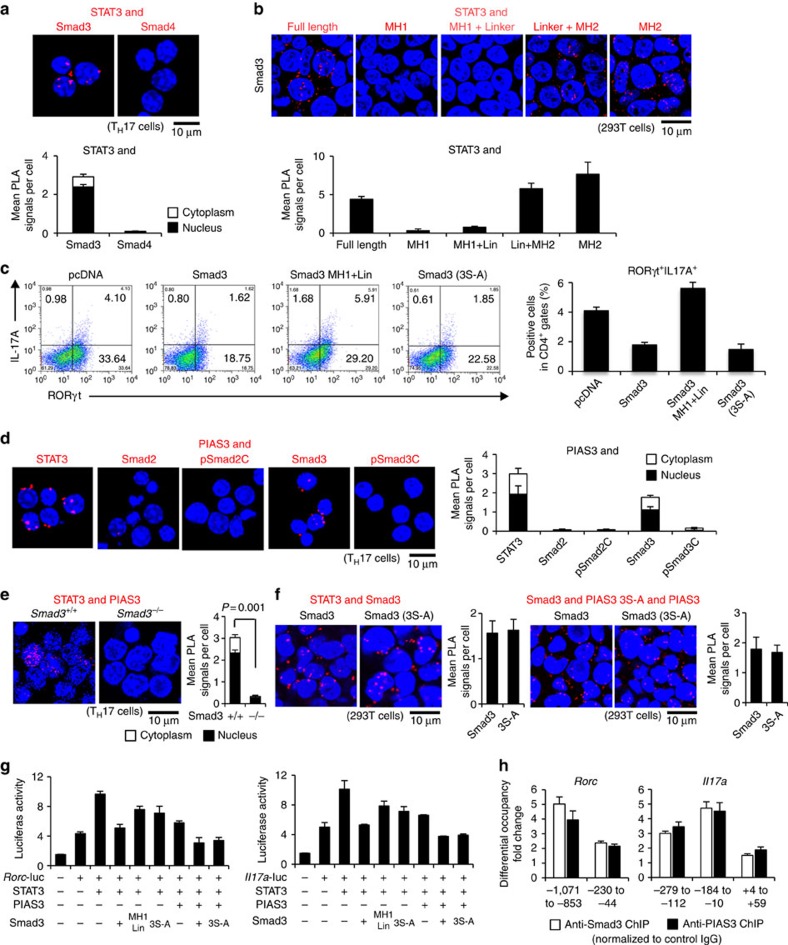
C-terminally unphosphorylated Smad3 recruits PIAS3 to act as a transcription co-repressor of STAT3 in T_H_17 differentiation. Interactions of endogenous proteins in T_H_17 cells and exogenous proteins in 293T cells were determined using PLA. PLA signals (**a**,**b**,**d**–**f**) were quantified using the BlobFinder software (scale bars, 10 μm; nucleus, black; cytoplasm, white, *n*=10 fields). (**a**) Endogenous interactions between Smad3/Smad4 and STAT3 in T_H_17 cells. (**b**) Effects of truncated mutations in Smad3 on the interaction with STAT3 in 293T cells. (**c**) Flow cytometry analyses of IL-17A^+^RORγt^+^CD4^+^ T cells transduced with the indicated DNA constructs using Nucleofector (*n*=4). (**d**) Endogenous interactions between PIAS3 and STAT3/Smad2/Smad3 in T_H_17 cells. (**e**) Endogenous interaction between PIAS3 and STAT3 in *Smad3*^*+/+, −/−*^ T_H_17 cells. (**f**) Effects of Smad3 C-terminal mutation on the interaction with STAT3 (left) or the interaction with PIAS3 (right) in 293T cells. (**g**) Effects of PIAS3, Smad3 MH2 deletion and Smad3 C-terminal mutation on STAT3-induced activation of the *Rorc* promoter and the *Il17a* promoter constructs transfected in T_H_17 cells were determined using the luciferase assay. (**h**) Binding of Smad3 (white) and PIAS3 (black) to the *Rorc* and the *Il17a* promoter regions in T_H_17 cells was determined using ChIP (differential occupancy fold changes). A representative of six (**a**), three (**b**,**f**,**g**), four (**c**,**d**) or two (**e**,**h**) independent experiments is shown. Each experiment (**g**,**h**) was performed in triplicate (*n*=3). Data are mean+s.d. or mean+s.d. with *P* values (**e**, unpaired Student's *t*-test).

We investigated the mechanism how Smad3 functions as a transcription co-repressor of STAT3. PLA confirmed the endogenous close proximity between Smad3 and STAT3 in T_H_17 cells (Fig. 4a). Unlike R-Smads, Smad4 did not interact with STAT3 (Fig. 4a). Although STAT5 and STAT3 oppositely regulate T_H_17 differentiation by binding the multiple common sites across the locus encoding IL-17 (ref. 27), neither Smad2 nor Smad3 interacted with STAT5 (Supplementary Fig. 11). Furthermore, interactions between Smad2/3 and STAT3 were as significant as the established interaction controls: pSmad2/3C-Smad4 (refs 9, 10) and Smad2/3–RORγt^18^,^21^ (Supplementary Fig. 12). PLA and immunoprecipitation of 293T cells transfected with the various deletion mutants of Smad3 showed that Smad3 MH2 deletion mutants (MH1 and MH1+L)^29^ failed to bind with STAT3 (Fig. 4b and Supplementary Fig. 13). Thus, the MH2 domain is required for Smad3 to bind STAT3.

PIAS3 belongs to the mammalian protein inhibitor of activated STAT (PIAS) protein family, which represses STAT3-dependent transcriptional activation by blocking the DNA-binding activity of STAT3, regardless of its small ubiquitin-like modifier-E3 ligase activity^32^. Overexpression of Smad3, the deletion mutant lacking MH2 domain or the C-terminal mutant in T_H_17 cells show that the Smad3 MH2 domain, but not the C-terminal SSXS motif, is functionally responsible for the suppression of T_H_17 differentiation (Fig. 4c). Because PIAS3 interacts with Smad3 at its C-terminal domain^33^, we examined whether Smad3 recruits PIAS3 to repress STAT3-induced transcription of the *Rorc* and *Il17a* genes. PIAS3 showed the close proximity with both STAT3 and Smad3, but not with C-terminally phosphorylated Smad3 (pSmad3C) or Smad2 in T_H_17 cells (Fig. 4d). STAT3–PIAS3 interaction was completely abolished in *Smad3*^−/−^ T_H_17 cells (Fig. 4e). A mutant of serine residues to alanine in the Smad3 SSXS motif, Smad3 (3S-A), was yet capable of binding with STAT3 and PIAS3 in 293T cells (Fig. 4f). Consistently, when co-transfected with PIAS3 in T_H_17 cells, Smad3 (3S-A) was able to suppress STAT3-induced activation of the *Rorc* and *Il17a* reporters (Fig. 4g). ChIP revealed that PIAS3 and Smad3, but not pSmad3C, bound to the same sites in the *Rorc* and *Il17a* promoters (Fig. 4h and Supplementary Fig. 14). Thus, C-terminal phosphorylation is not required for Smad3 to bind with STAT3 and PIAS3. Overexpression of PIAS3 suppressed T_H_17 differentiation, whereas knockdown of PIAS3 by siRNA abolished the binding of Smad3 to the *Rorc* and *Il17a* promoter regions, although T_H_17 differentiation was unaltered by knockdown of PIAS3 presumably because relatively predominant binding of Smad2 over Smad3 in the absence of PIAS3 transactivated the *Rorc* and *Il17a* genes (Supplementary Fig. 15). These data indicate that unphosphorylated Smad3C in cooperation with PIAS3 represses STAT3-induced transcription of the *Rorc* and *Il17a* genes.

### ERK phosphorylates Smad2 linker in T_H_17 differentiation

Previous studies have paid attention to C-terminal phosphorylation of R-Smads as TGF-β signalling mediators in T_H_17 differentiation^18^,^19^,^20^,^21^,^22^,^23^. However, pSmad2L (Ser255) and unphosphorylated Smad3 are not involved in the canonical C-terminally phosphorylated R-Smad/Smad4-mediated TGF-β signalling. Three clustered serine residues in the linker regions of Smad2 (Ser245/250/255) are the phosphorylation sites for MAPKs (ERK, JNK and p38)^10^,^11^,^12^,^13^,^34^. Because MAPKs are shared by TGF-β, IL-6 and TCR, we sought to identify the MAPK responsible for Smad2 linker phosphorylation in T_H_17 differentiation.

**Figure 5:**
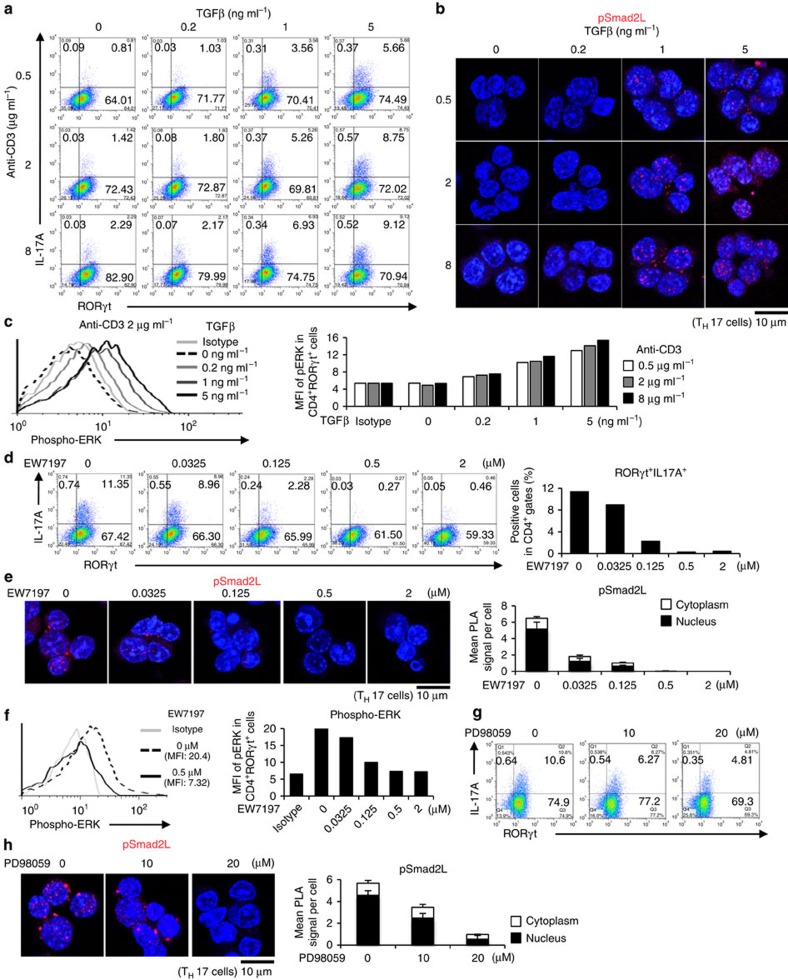
ERK induces Smad2 linker phosphorylation that facilitates T_H_17 differentiation. Purified CD4^+^ T cells were activated under T_H_17-polarizing condition with the indicated doses of TGF-β1 and plate-coated anti-CD3 antibody, or small molecule inhibitors (EW-7197, ALK5 inhibitor; PD98059, MEK inhibitor) for 3 days. (**a**) Flow cytometry analyses of IL-17A^+^RORγt^+^CD4^+^ T cells treated with TGF-β1 and plate-coated anti-CD3 antibody. (**b**) Endogenous expression of pSmad2L in T_H_17 cells treated with TGF-β1 and plate-coated anti-CD3 antibody was determined using PLA. (**c**) Flow cytometry analyses of phospho-ERK in T_H_17 cells treated with TGF-β1 and plate-coated anti-CD3 antibody. (**d**) Flow cytometry analyses of IL-17A^+^RORγt^+^CD4^+^ T cells treated with EW-7197. (**e**) Endogenous expression of pSmad2L in T_H_17 cells treated with EW-7197 was determined using PLA. (**f**) Flow cytometry analyses of phospho-ERK in T_H_17 cells treated with EW-7197. (**g**) Flow cytometry analyses of IL-17A^+^RORγt^+^CD4^+^ T cells treated with PD98059. (**h**) Endogenous expression of pSmad2L in T_H_17 cells treated with PD98059 was determined using PLA. The values of the mean fluorescence intensity (MFI) are shown in graphs. PLA signals (**b**,**e**,**h**) were quantified using the BlobFinder software (scale bars, 10 μm; nucleus, black; cytoplasm, white, *n*=10 fields). Data are representative of two (**a**–**h**) independent experiments. Data are mean+s.d.

Signal intensities of TGF-β, IL-6 and TCR have been reported to correlate with the extent of T_H_17 differentiation^3^,^4^,^16^. Therefore, we treated CD4^+^ T cells under T_H_17-polarizing condition with various concentrations of TGF-β, IL-6 and anti-CD3 antibody. We confirmed that higher doses of TGF-β1, anti-CD3 antibody and IL-6 induced more T_H_17 differentiation (Fig. 5a and Supplementary Figs 16a and 17a). Percentages of IL-17A^+^RORγt^+^CD4^+^ T cells were directly proportional to phosphorylation of Smad2L (Fig. 5b and Supplementary Figs 16b and 17b) and ERK, but not to the phosphorylation of JNK or p38 (Fig. 5c and Supplementary Figs 18 and 19).

To confirm whether TβRI-mediated phosphorylation of Smad2L is required for T_H_17 differentiation, we treated CD4^+^ T cells under T_H_17-polarizing condition with specific inhibitors against TβRI^35^ at the doses that maintain cell viability (Supplementary Fig. 20). A potent selective ATP-competitive inhibitor of TβRI kinase (activin receptor-like kinase5: ALK5), EW-7197 (refs 35, 36) completely suppressed T_H_17 differentiation at the dose of 0.5 μM (Fig. 5d). Treatment with EW-7197 suppressed pSmad2L (Fig. 5e) and phosphorylation of ERK, but not phosphorylation of JNK and p38 (Fig. 5f and Supplementary Fig. 21). One of the prototype ALK5 inhibitors, SB-505124, inhibits TGF-β-induced activation of MAPKs without altering ALK5-independent MAP kinase pathways^37^. A more highly selective ALK5 inhibitor, EW-7197, does not directly inhibit MEK1 and ERK1 (ref. 35). Therefore, inhibitory effect of EW-7197 on ERK phosphorylation is ALK5-specific. Culture media containing IL-6, IL-23 and IL-1β is sufficient to induce T_H_17 in the absence of TGF-β (ref. 38). However, EW-7197 inhibited, whereas TGF-β1 enhanced T_H_17 differentiation along with ERK phosphorylation even under this culture condition (Supplementary Fig. 22). These results suggest that TGF-β-TβRI signal phosphorylates ERK and Smad2L in T_H_17 cells.

To confirm whether ERK-mediated phosphorylation of Smad2L is required for T_H_17 differentiation, we next treated CD4^+^ T cells under T_H_17-polarizing condition with specific inhibitors against MAPKs at the doses that maintain cell viability (Supplementary Figs 23 and 24). A MEK inhibitor PD98059 suppressed T_H_17 differentiation in a dose-dependent manner (Fig. 5g and Supplementary Fig. 23), whereas a JNK inhibitor SP600125 or p38 inhibitor SB203580 did not affect T_H_17 differentiation (Supplementary Fig. 24). PD98059 showed the similar effects with EW-7197 on pSmad2L (Fig. 5h). Specific inhibition of MAP kinase by the corresponding inhibitor was confirmed (Supplementary Fig. 25). Taken together, ERK-mediated Smad2 linker phosphorylation is responsible for T_H_17 differentiation and the concentrations of TGF-β, TCR and IL-6 determine the intensities of Smad2 linker phosphorylation and the extent of T_H_17 differentiation.

### R-Smad–STAT3 interactions balance T_H_17 differentiation

**Figure 6:**
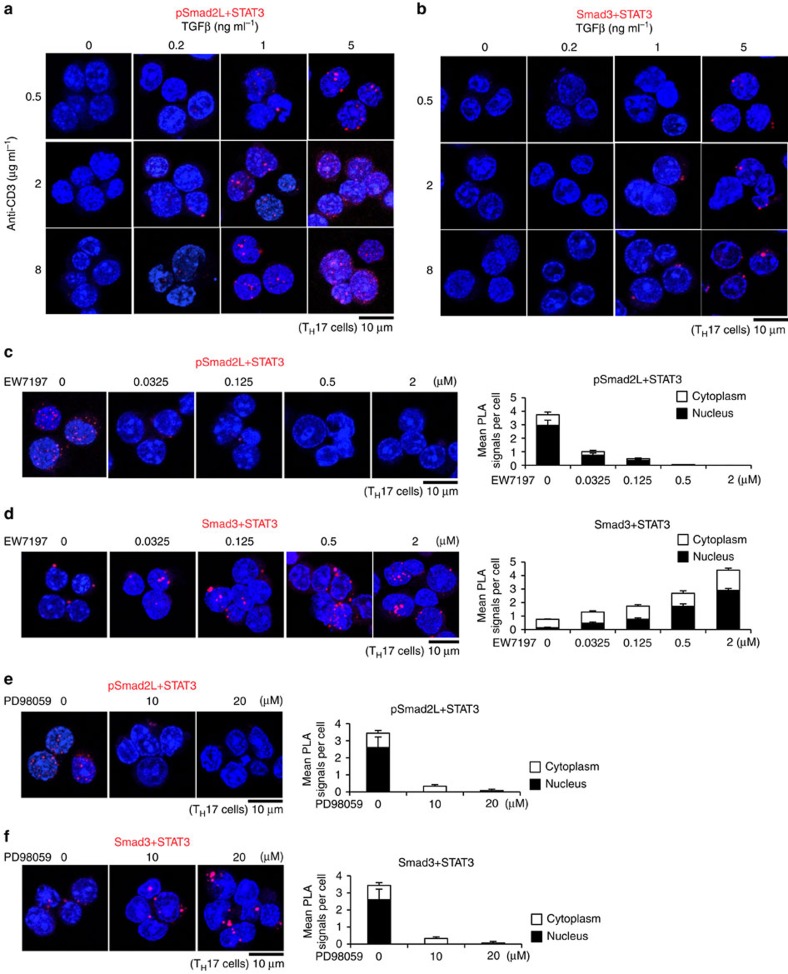
R-Smad–STAT3 interaction balances correlate with T_H_17 differentiation. Purified CD4^+^ T cells were activated under T_H_17-polarizing condition with the indicated doses of TGF-β1 and plate-coated anti-CD3 antibody, or small molecule inhibitors (EW-7197, ALK5 inhibitor; PD98059, MEK inhibitor) for 3 days. Interactions of endogenous proteins in T_H_17 cells were determined with PLA. PLA signals (**a**–**f**) were quantified using the BlobFinder software (scale bars, 10 μm; nucleus, black; cytoplasm, white, *n*=10 fields). (**a**) pSmad2L and STAT3, (**b**) Smad3 and STAT3 in T_H_17 cells treated with the indicated doses of TGF-β1 and plate-coated anti-CD3 antibody. (**c**) pSmad2L and STAT3, (**d**) Smad3 and STAT3 in T_H_17 cells treated with the indicated doses of EW-7197. (**e**) pSmad2L and STAT3, (**f**) Smad3 and STAT3 in T_H_17 cells treated with the indicated doses of PD98059. Data are representative of two independent experiments. Data are mean+s.d.

We next examined the effects of intensities and inhibitions of TGF-β/IL-6/TCR signals on the interactions of STAT3 with pSmad2L or unphosphorylated Smad3C in T_H_17 cells. Higher doses of TGF-β1, IL-6 and anti-CD3 antibody significantly upregulated pSmad2L–STAT3 interactions with little changes in Smad3–STAT3 interactions (Fig. 6a,b and Supplementary Fig. 26). By contrast, treatments with EW-7197 or PD98059 significantly downregulated pSmad2L–STAT3 interactions (Fig. 6c,e), whereas upregulated Smad3–STAT3 interactions (Fig. 6d,f). Interactions of pSmad2L and STAT3 were directly proportional, whereas interactions of unphosphorylated Smad3C and STAT3 were inversely proportional to T_H_17 differentiation of EW-7197- or PD98059-treated CD4^+^ T cells (Figs 5d,g and 6c–f). These data suggest that the balances between STAT3-interacting pSmad2L and STAT3-interacting unphosphorylated Smad3C determine the extent of T_H_17 differentiation.

**Figure 7:**
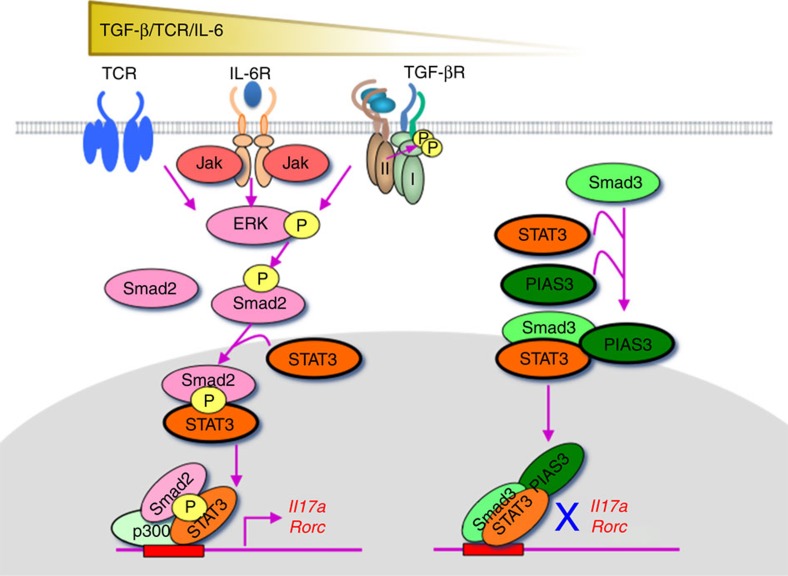
TGF-β R-Smads, Smad2 and Smad3, oppositely regulate T_H_17 differentiation as transcription cofactors of STAT3. ERK-phosphorylated Smad2L (S255)/STAT3/p300 activates, whereas unphosphorylated Smad3C/STAT3/PIAS3 represses the transcription of the *Rorc* and *Il17a* genes.

In summary, the TGF-β/IL-6/TCR–pERK–pSmad2L (Ser255) axis is the positive regulator, whereas unphosphorylated Smad3C–PIAS3 complex is the negative regulator of STAT3-induced transcriptional processes for T_H_17 differentiation (Fig. 7).

## Discussion

We discovered that Smad2 and Smad3 oppositely regulated STAT3-induced T_H_17 differentiation through the novel direct signalling networks. Transmodulation between the SMAD and STAT signalling pathways balances the interplay between TGF-β and various cytokines. Indirect crosstalk between SMAD and STAT was first reported as the inhibition of Smad3/4-mediated TGF-β signalling by Jak1-STAT1-mediated interferon (IFN)-γ signalling via induction of the inhibitory Smad, Smad7, which prevents TβRI-induced C-terminal phosphorylation of Smad3 (ref. 39). Direct crosstalk between SMAD and STAT was discovered as the synergistic signalling of leukaemia inhibitory factor and bone morphogenic protein-2, one of the TGF-β superfamily cytokines, via the STAT3–Smad1 complex bridged by p300 in fetal neural cells^40^. Direct crosstalk between Smad3 and STAT3 was reported as the augmentation of IL-6-STAT3-mediated transactivation by TGF-β via interaction of the STAT3–pSmad3C complex bridged by p300 in hepatoma cells^41^. This study clarified the mechanisms whereby R-Smads–STAT3 networks modulate T_H_17 differentiation; pSmad2L (Ser255) serves as STAT3 co-activator in combination with p300, a co-activator of various transcription factors including both Smads and STAT3 (refs 27, 31), whereas unphosphorylated Smad3C serves as the STAT3 co-repressor in combination with PIAS3, a negative regulator of STAT3-induced transcription^32^. The preceding reports and our findings indicate that SMAD–STAT signalling networks are highly cell-type-specific and context-dependent. Because of the relatively low DNA-binding affinity of Smad3 and lack of DNA-binding ability of Smad2, they interact with a wide variety of DNA-binding proteins to co-regulate the target genes. Recently, genome-wide transcriptome analyses have elucidated the diverse regulatory networks of Smad2/3 with cell-type-specific master transcription factors and/or DNA-binding cofactors in variety of cells^42^. The thorough iterative approach to delineate the T_H_17 global transcriptional regulatory network shows that STAT3 works as one of the key activators of the initial transcriptional programme, RORγt works as an expression modulator and Smad3 is the negative regulators^43^. It is noteworthy that a histone demethylase, JMJD3 (KDM6B) regulates the expression of numerous targets of RORγt and STAT3 (ref. 43) because JMJD3 causes a loss of the H3K27me3-repressive epigenetic mark by interacting with R-Smads at their target sites^42^. Therefore, it is possible that Smad2 may interact with JMJD3 to induce active chromatin state for T_H_17 regulation in the same manner with Nodal-Smad2/3 signalling in embryonic development^44^ (Supplementary Fig. 8). Further studies are required to elucidate the details of divergent context-dependent SMAD–STAT signalling networks implicated by genome-wide transcriptome analyses.

We further uncovered the novel roles of R-Smads with noncanonical phosphorylation status in networking with STAT3: linker phosphorylated Smad2 as a STAT3 co-activator and unphosphorylated Smad3 as a STAT3 co-repressor. Serine/threonine-rich R-Smad linker regions contain multiple phosphorylation sites by proline-directed protein kinases such as MAPKs, glycogen synthase kinase 3 and cyclin-dependent kinase family^11^,^12^,^13^. Linker residues Ser245/250/255, Thr220 in Smad2 and Ser204/208/213, Thr179 in Smad3 are the sites for phosphorylation^12^,^13^. Three clustered serine residues are preferred phosphorylation sites for ERK, JNK and p38 in response to receptor tyrosine kinases and proinflammatory cytokines, whereas threonine residues are preferred phosphorylation sites for cyclin-dependent kinase family in response to TGF-β. TβRI possesses the intrinsic tyrosine kinase activity to directly induce activation of MAPK pathways and subsequent phosphorylation of R-Smad linker residues in addition to the serine/threonine kinase activity to phosphorylate R-Smads in their conserved C-terminal SSXS motif^5^,^11^. Mitogens and hyperactive Ras induce ERK-mediated linker phosphorylation of Smad2 at Ser245/250/255/Thr220 and Smad3 at Ser204/208/Thr179 (refs 12, 13). Therefore, roles of R-Smad linker phosphorylation in carcinogenesis have been investigated intensively^11^,^12^,^13^. Central role of ERK in TCR signals^15^,^24^ suggests the important roles of R-Smad linker phosphorylation in T-cell signalling network. Thus far, MEKK2/3-ERK1/2 signalling has been reported to induce pSmad3L, which negatively regulates canonical TGF-β signalling for T_H_17 differentiation^45^. Because we found that Smad3 linker region was not involved in STAT3-induced T_H_17 differentiation (Fig. 4b), the mechanisms how pSmad3L regulates T_H_17 differentiation are independent of STAT3. By contrast to phosphorylated Smads, very little has been known about physiological functions of unphosphorylated R-Smads. It has been reported that PIAS3 enhances TGF-β-induced transcriptional activity of C-terminally phosphorylated Smad3 by recruiting p300 and CBP in COS and 293T cells^33^. By contrast, we discovered that unphosphorylated Smad3C due to less TGF-β signalling was required for PIAS3 to function as a co-repressor of STAT3 (ref. 32). Recent genome-wide studies implicate that unphosphorylated Smad3 may bind to some cell-type-specific transcription factors in both TGF-β-dependent and TGF-β-independent manners^42^,^46^,^47^. Our finding shed light on as-yet-unrecognized functions of unphosphorylated Smad3 as a transcription cofactor.

The discovery of a new proinflammatory effector T-cell subset, T_H_17, revised the functions of TGF-β, which had been long considered as the most potent immunosuppressive cytokine. TGF-β has been identified as the requisite factor for T_H_17 differentiation in combination with IL-6 and other proinflammatory cytokines such as IL-21, IL-23, IL-1β and tumour-necrosis factor-α (TNF-α)^3^,^4^,^6^. However, as functions of TGF-β have been frequently described as dual, bidirectional, pleiotropic, complex or contextual^5^,^9^,^10^,^11^,^12^,^13^, the roles of TGF-β in T_H_17 differentiation have become controversial^18^,^19^,^20^,^21^,^22^,^23^. Requirement of TGF-β for T_H_17 differentiation remains contradictory, indispensable^4^ or dispensable^38^. Our results provide explanation for these conflicting reports. TGF-β ligand-independent T_H_17 differentiation is possible because Smad2 linker phosphorylation could be induced by ERK signals downstream of the IL-6 receptor and TCR. However, significantly more effective inhibitory effect of the ALK5 inhibitor than that of a MEK inhibitor suggests that the TβRI–pERK–pSmad2L axis is more efficient than the non-TGF-β growth-stimulatory signal-pERK-pSmad2L axis for T_H_17 differentiation. Nonetheless, the report showing the dispensability of TGF-β demonstrates that TGF-β induces significantly more T_H_17 differentiation^38^. Likewise, our results provide explanations for the discrepancies in the reported roles of Smads in T_H_17 differentiation^18^,^19^,^20^,^21^,^22^,^23^. Our data are consistent with the previous reports showing that Smad2 is a positive regulator and Smad3 is a negative regulator of T_H_17 differentiation, although the mechanisms of actions are distinct^18^,^19^,^20^,^21^. It has been reported that TGF-β signalling via Smad2 indirectly induced STAT3 phosphorylation by inducing the expression of mRNA and protein of IL-6Rα (ref. 19); however, we could not confirm the differences in IL-6Rα mRNA expression in our systems (Supplementary Fig. 7). It has been reported that Smad3 interacted with RORγt and decreased its transcriptional activity^21^. We confirmed that not only Smad3 but also Smad2 interacted with RORγt (Supplementary Fig. 12). Whether RORγt forms the complex with STAT3 and R-Smads remains to be determined. Our data also suggest that the signalling intensity balances of TCR, co-stimulation, IL-6, TGF-β and other cytokines could yield the seeming dispensability of R-Smads^22^,^23^ because of their opposing effects. The signalling balances between TβRI-PKCα-mediated C-terminal phosphorylation of R-Smads^48^ and pERK-pSmad2L may be also crucial for T_H_17 differentiation.

An ALK5 inhibitor is efficacious against a mouse type II collagen antibody-induced arthritis model^49^. Our results of a CIA model showed the promoting role of pSmad2L at Ser255 and the suppressive role of unphosphorylated Smad3 in the arthritogenic T_H_17 differentiation. Therefore, inhibiting phosphorylation of Smad2 linker or Smad3 C-terminus may have therapeutic utility for RA and various T_H_17-related inflammatory diseases. Considering the crucial roles of SMADs and STATs in cell regulation, homeostasis and the pathogenesis of various diseases such as infection, cancers, fibrosis and inflammation, our findings will lead to the elucidation of cytokine signalling networks in various settings. In summary, we show the novel signalling networks of R-Smads and STAT3 for T_H_17 differentiation, which revise the classical linear signalling cascades^50^.

## Methods

### Mice

*Smad2*^*3loxp/3loxp*^ mice targeting exons 9 and 10 (ref. 51), *Smad3*^*ex8/ex8*^ mice targeting exon 8 (ref. 52) were generated as described on Sv129 × C57BL/6J background and backcrossed to C57BL/6J mice (Nihon SLC) for eight generations. For *in vitro* experiments, *Smad3*^*ex8/ex8*^ mice were backcrossed to C57BL/6J background for four generations. We used *Smad3*^*+/−*^ mice because *Smad3*^*−/−*^ mice develop osteoarthritis, bone malformation^53^ and impaired mucosal immunity^52^, and the embryonic lethality of *Smad3*^*−/−*^ mice in the C57BL/6 background was extremely high (Supplementary Fig. 27), similarly with *Tgf-β1*^*−/−*^ mice^54^. *Cd4Cre* transgenic mice^55^ were purchased from Jackson Laboratories. *Mx-1Cre* transgenic mice^56^ were kindly provided by Dr Masayuki Yamamoto (Tohoku University, Japan). For *Mx-1Cre* mice at 2–3 weeks of age, gene deletion was induced by intraperitoneal injections of 250 μg polyI:C dissolved in sterile saline at 2-day intervals for a total of three injections^56^. For immunophenotyping, spleens and superficial lymph nodes (cervical, axillary, brachial and inguinal) from the female mice aged between 12 and 16 weeks of age were used (age- and sex-matched, no randomized). All animals were maintained and used for experiments according to the ethical guidelines for animal experiments and the safety guidelines for gene manipulation experiments at the Konkuk University, Republic of Korea, University of Tsukuba, Japan, Tokyo Medical University, Japan under approved animal study protocols.

### CIA

For induction of CIA, we used the immunization protocol for C57BL/6 strain (H-2b)^57^. Briefly, mice aged between 8 and 10 weeks of age were injected intradermally at several sites into the base of the tail and back with type II collagen (Sigma-Aldrich, Cat. no. C9301) emulsified in complete Freund adjuvant: incomplete Freund adjuvant (GIBCO), heat-killed *mycobacterium tuberculosis* (Difco Laboratories) on day −21 and the same injection was repeated on day 0 (Fig. 1a and Supplementary Fig. 2). Arthritis development in each paw was scored by macroscopic evaluation^58^ as follows: (0) no change, (1) erythema and mild swelling confined to the ankle, (2) erythema and mild swelling from the ankle to midfoot, (3) moderate swelling and (4) severe swelling. The maximum score per mouse is 16. The investigators (M.M., K.S., S.N. and J.S.H.) were blinded to the genotypes. Ten to twenty mice/genotype were used (Fig. 1 legend and Supplementary Fig. 2 legend). Mice were dissected 2 weeks after the second immunization to evaluate the draining lymph nodes (popliteal, inguinal, axillary and brachial).

### Histological analysis

Paws from collagen-immunized mice were harvested, fixed in 10% neutral-buffered formalin, decalcified, dehydrated with 70% ethanol, embedded in paraffin and sectioned at 3 μm. Sections were stained with haematoxylin and eosin, toluidine blue or safranin O. For immunohistochemistry, sections were stained with rat anti-CD4 (Abcam, Cat. no. ab25475, 1:50), rabbit anti-RORγt (Abcam, Cat. no. ab78007, 1:50) and rabbit anti-IL-17A (Abcam, Cat. no. ab79056, 1:50) antibodies. Slides were observed using an optical microscope, DM5000B (Leica).

### Flow cytometry analyses

Fluorophore-conjugated antibodies were purchased from BD Pharmingen and eBioscience. CD16/32 were blocked by Fc-Block (BD Pharmingen, Cat. no. 553142) and isotype-matched control antibodies were used in each experiment. For cytokine intracellular staining, cultured cells or freshly isolated cells from CIA mice were treated with 5 ng ml^−1^ of phorbol-12-myristate 13-acetate (Sigma-Aldrich) and 500 ng ml^−1^ of ionomycin (Sigma-Aldrich) in the presence of GolgiPlug (BD Pharmingen) for the last 4 h of culture. For intracellular staining, cultured cells were fixed with the Cytoperm/Cytofix kit (BD Pharmingen). For Foxp3 staining, the Foxp3 staining kit (eBioscience, Cat. no. 00-5523-00) was used. Stained cells were acquired and analysed using LSR II (BD) and FlowJo software (Tree Star Inc.).

### T-cell stimulation *in vitro*

CD4^+^ T cells from superficial lymph nodes and spleens were enriched using T-cell enrichment columns (R&D Systems) and MACS system (Miltenyi Biotech). Purity was confirmed as >90% using LSR II. In some experiments, CD44^low^CD62L^high^CD4^+^ T cells were sorted with FACSAria (BD) and the purity was >98%. Purified CD4^+^ T cells were stimulated by plate-coated anti-CD3 (2.0 μg ml^−1^; BD Pharmingen, Cat. no. 553057) and soluble anti-CD28 antibodies (5.0 μg ml^−1^; BD Pharmingen, Cat. no. 553294) with mIL-6 (50 ng ml^−1^), TGF-β1 (1 ng ml^−1^; Peprotech), anti-mouse IL-4 (10 μg ml^−1^; Biolegend, Cat. no. 504108) and anti-mouse IFN-γ antibodies (10 μg ml^−1^; BioLegend, Cat. no. 505812) in 10% FCS RPMI 1640 media supplemented with penicillin and streptomycin (HyClone) for T_H_17 differentiation for 3–4 days^3^. In some experiments, Purified CD4^+^ T cells were stimulated by the indicated doses of plate-coated anti-CD3, TGF-β1 and mIL-6, or by plate-coated anti-CD3 (10.0 μg ml^−1^; BD Pharmingen, Cat. no. 553057), soluble anti-CD28 antibodies (5.0 μg ml^−1^; BD Pharmingen, Cat. no. 553294), IL-1β (10 ng ml^−1^; Peprotech), IL-23 (10 ng ml^−1^; Peprotech) and the neutralizing antibodies described above, or by the indicated doses of various small molecule inhibitors: EW-7197 (ALK5 inhibitor) from Dr Dae-Kee Kim, PD98059 (MEK inhibitor), SP600125 (JNK inhibitor) and SB203580 (p38 inhibitor; Sigma-Aldrich). STAT3 siRNA (Dharmacon), PIAS3 siRNA (Santa Cruz), DNA constructs: Smad2, Smad2 (S255A), Smad3, Smad3 (3S-A) and Smad3 (MH1+L) from Dr Koichi Matsuzaki and Dr Takeshi Imamura, PIAS3 (Addgene, submitted by Shuai) and pcDNA or control RNA were transfected to purified CD4^+^ T cells using the 4D-Nucleofector and Amaxa Mouse T-cell Nucleofector kit (Lonza) before the cell culture.

### RNA isolation and quantitation of mRNA using real-time RT–PCR

Total RNA was extracted using Trizol according to the manufacturer's instructions (Invitrogen). RNA was reverse-transcribed with the cDNA reverse transcription kit (Invitrogen). Amount of cDNA was quantitated with SYBR green (Applied Biosystems) real-time PCR using ABI 7900 and ABI 7300 machines (Applied Biosystems). The primers are described in Supplementary Table 1.

### Western blot analysis and immunoprecipitation

293T cells (ATCC-CRL-3216) were transfected using PEI with STAT3 (Addgene, submitted by J. Darnell), FLAG-tagged Smad2 (full length, MH1, MH1+Linker, MH2+Linker, MH2, Y220V, S245A, S250A, S255A) and FLAG-tagged Smad3 (full length, MH1, MH1+Linker, MH2+Linker, MH2, 3S-A) from Dr Koichi Matsuzaki and Dr Takeshi Imamura. Cells were lysed with lysis buffer (PBS containing 0.5% Triton X-100, 20 mM HEPES (pH 7.4), 150 mM NaCl, 12.5 mM β-glycerol phosphate, 1.5 mM MgCl2, 10 mM NaF, 2 mM dithiothreitol, 1 mM NaOV, 2 mM EGTA, 1 mM phenylmethylsulphonyl fluoride (PMSF) and protease inhibitor cocktail) were electrophoresed on 10% SDS–polyacrylamide gel and transferred to the polyvinylidene difluoride (PVDF) membrane, and probed with antibodies against phospho-Smad2 (Abcam, Cat. no. ab53100, 1:1,000 dilution), phospho-Smad3 (Abcam, Cat. no. ab51451, 1:1,000 dilution), Smad2 (Santa Cruz, Cat. no. sc-101153, 1:1,000 dilution), Smad3 (Santa Cruz, Cat. no. sc-101154, 1:1,000 dilution), Smad4 (Santa Cruz, Cat. no. sc-7966, 1:1,000 dilution) and β-actin (Santa Cruz, Cat. no. sc-7210, 1:10,000 dilution). Blots were visualized using an electrochemiluminescence kit (GE Healthcare).

For immunoprecipitation, the lysates were cleared using centrifugation at 16,000*g* for 10 min, incubated with protein A/G agarose beads and with anti-STAT3 antibody (Santa Cruz, Cat. no. sc-7179, 2.0 μg per 1 ml of cell lysate) at 4 °C for 12–16 h. The beads were washed three times with lysis buffer and immunoprecipitates were separated from the beads by adding 2 × sample buffer and boiled. SDS–PAGE-separated immunoprecipitates were transferred on PVDF membranes. The membranes were denatured with denaturation buffer containing 6 M guanidine chloride, 20 mM Tris (pH 7.5), 100 mM PMSF and 5 mM β-mercaptoethanol at 4 °C for 30 min and washed three times with TBST. The membranes were blocked with 5% BSA and incubated with anti-FLAG antibody (Biomol, Cat. no. ADI-SAB-410-0100, 1:1,000 dilution). 293T cells (ATCC-CRL-3216) were confirmed to be mycoplasma-negative using the e-Myco plus Mycoplasma PCR Detection Kit (iNtRON Biotechnology, Cat. no. 25237).

### PLA

CD4^+^ T cells cultured in T_H_17 condition for 3–5 days or 293T cells (ATCC-CRL-3216) transfected with various constructs were fixed on the slides by 3.7% formaldehyde in PBS. The slides were washed, permeabilized by 0.1% Triton X-100 in TBS and blocked by 0.5% BSA. PLA was performed using the Duolink II Fluorescence kit (OLINK) using the rabbit antibodies (1:50 dilution) against: STAT3 (Cell Signaling Technology, Cat. no. 12640), phospho-STAT3 Y705 (Cell Signaling Technology, Cat. no. 9145), phospho-STAT3 S727 (Cell Signaling Technology, Cat. no. 9134), Smad2 (Cell Signaling Technology, Cat. no. 5339), phospho-Smad2C S465/467 (Cell Signaling Technology, Cat. no. 3101), phospho-Smad2L S245/250/255 (Cell Signaling Technology, Cat. no. 3104), Smad3 (Cell Signaling Technology, Cat. no. 9523), phospho-Smad3C S423/425 (Cell Signaling Technology, Cat. no. 9520), RORγt (Abcam, Cat. no. ab78007), PIAS3 (Santa Cruz, Cat. no. sc-14017), Flag (Biomol, Cat. no. ADI-SAB-410-0100) and phospho-Smad3L S208/213 (IBL, Cat. no. JP28029), mouse antibodies (1:50 dilution) against: Smad2/3 (Santa Cruz, Cat. no. sc-133098), Smad4 (Santa Cruz, Cat. no. sc-7966), STAT3 (Santa Cruz, Cat. no. sc-8019) and p300 (Santa Cruz, Cat. no. sc-48343). Target-specific rabbit primary antibodies and the secondary antibodies conjugated with oligonucleotides: PLA probe anti-rabbit PLUS (Sigma-Aldrich, Cat. no. DUO92002) and PLA probe anti-rabbit MINUS (Sigma-Aldrich, Cat. no. DUO92005) or PLA probe anti-mouse PLUS (Sigma-Aldrich, Cat. no. DUO92001) and PLA probe anti-mouse MINUS (Sigma-Aldrich, Cat. no. DUO92004) were used for single recognitions. Two primary antibodies raised in different species and the secondary antibodies conjugated with oligonucleotides: PLA probe anti-rabbit PLUS and PLA probe anti-mouse MINUS were used for double recognitions. After incubation of the slides with Blocking Solution for 30 min at 37 °C, they were incubated with primary antibodies diluted in the Antibody Diluent overnight at 4 °C, in PLA probe solution for 1 h at 37 °C and in Ligation-Ligase solution for 30 min at 37 °C with washing with Wash Buffer A in the interim of each step. The slides were incubated in Amplification-Polymerase solution for 100 min at 37 °C and then washed in Wash Buffer B. The nucleus was stained with DAPI. Then, the slides were dried at room temperature in the dark. Slides were observed using a confocal microscope, LSM700 (Carl Zeiss). PLA signals were quantified using the BlobFinder software (Centre for Image Analysis, Uppsala University).

### Luciferase assay

The 2,000-bp promoter region of RORγt was generated using PCR from genomic C57BL/6 DNA using primers described in Supplementary Table 2. Products were verified by sequencing and were subcloned into pGL4 firefly luciferase construct (Promega) using NheI, EcoRV sites and XhoI, HindIII sites, respectively. The pGL4 mIL-17 2-kb promoter construct was from Addgene (submitted by W. Strober). The promoter constructs in various combinations with Flag-tagged STAT3 (Addgene, submitted by J. Darnell), Flag-tagged Smads, Flag-tagged Smad mutants, haemagglutinin-tagged p300, Flag-tagged PIAS3 (Addgene, submitted by Shuai) or empty pcDNA3 plasmid were co-transfected with control TK-pRL Renilla plasmid using PEI for 293T cells or using the 4D-Nucleofector and Amaxa Mouse T-cell Nucleofector kit (Lonza) for T_H_17 cells. Six hours after transfection, 293T cells (ATCC-CRL-3216) were lysed for the measurement using luminometer. CD4^+^ T cells were transfected before the cell culture under T_H_17-polarizing condition for 4 days.

### ChIP

Chromatin was prepared from 1 × 10^7^ CD4^+^ T cells isolated from C57BL/6 mice, *Cd4Cre;Smad2*^*fl/fl*^ mice, *Smad3*^*−/−*^ mice and the littermate control mice under T_H_17-polarizing condition for 3–4 days. Immunoprecipitation was performed with antibodies (1:50 dilution) against Smad2 (Cell Signaling Technology, Cat. no. 5339), Smad3 (Cell Signaling Technology, Cat. no. 9523), phospho-Smad3C S423/425 (Cell Signaling Technology, Cat. no. 9520), Smad4 (Santa Cruz, Cat. no. sc-7966), STAT3 (Santa Cruz, Cat. no. sc-7179), tri-methyl histone H3 Lys4 (Cell Signaling Technology, Cat. no. 9751), tri-methyl histone H3 Lys27 (Cell Signaling Technology, Cat. no. 9733), acetyl histone H3 Lys23 (Millipore, Cat. no. 17–10112) and PIAS3 (Santa Cruz) using ChIP kit (Cell Signaling Technology) according to the manufacturer's protocol. Immunoprecipitated DNA released from the crosslinked proteins was quantitated with real-time PCR using the primers (Supplementary Table 3) and was normalized to input DNA.

### Statistical analyses

Statistical analysis was performed using analysis tools on the VassarStats Statistical Computation site (http://vassarstats.net/) and Excel. Data were analysed using the parametric unpaired Student *t*-test, or two-way analysis of variance test for CIA scoring.

## Additional information

**How to cite this article:** Yoon, J.-H. *et al*. Phosphorylation status determines the opposing functions of Smad2/Smad3 as STAT3 cofactors in T_H_17 differentiation. *Nat. Commun*. 6:7600 doi: 10.1038/ncomms8600 (2015).

## Supplementary Material

Supplementary Information Supplementary Figures 1-27 and Supplementary Tables 1-3
