# Supplementary material for: Phosphorylation status determines the opposing functions of Smad2/Smad3 as STAT3 cofactors in TH17 differentiation
Source: Nat Commun. 2015 Jul 21;6:7600. doi: 10.1038/ncomms8600 (PMC4518312; doi:10.1038/ncomms8600)
Supplement: Supplementary Information — Supplementary Figures 1-27 and Supplementary Tables 1-3 [file ncomms8600-s1.pdf]

Supplementary Figures

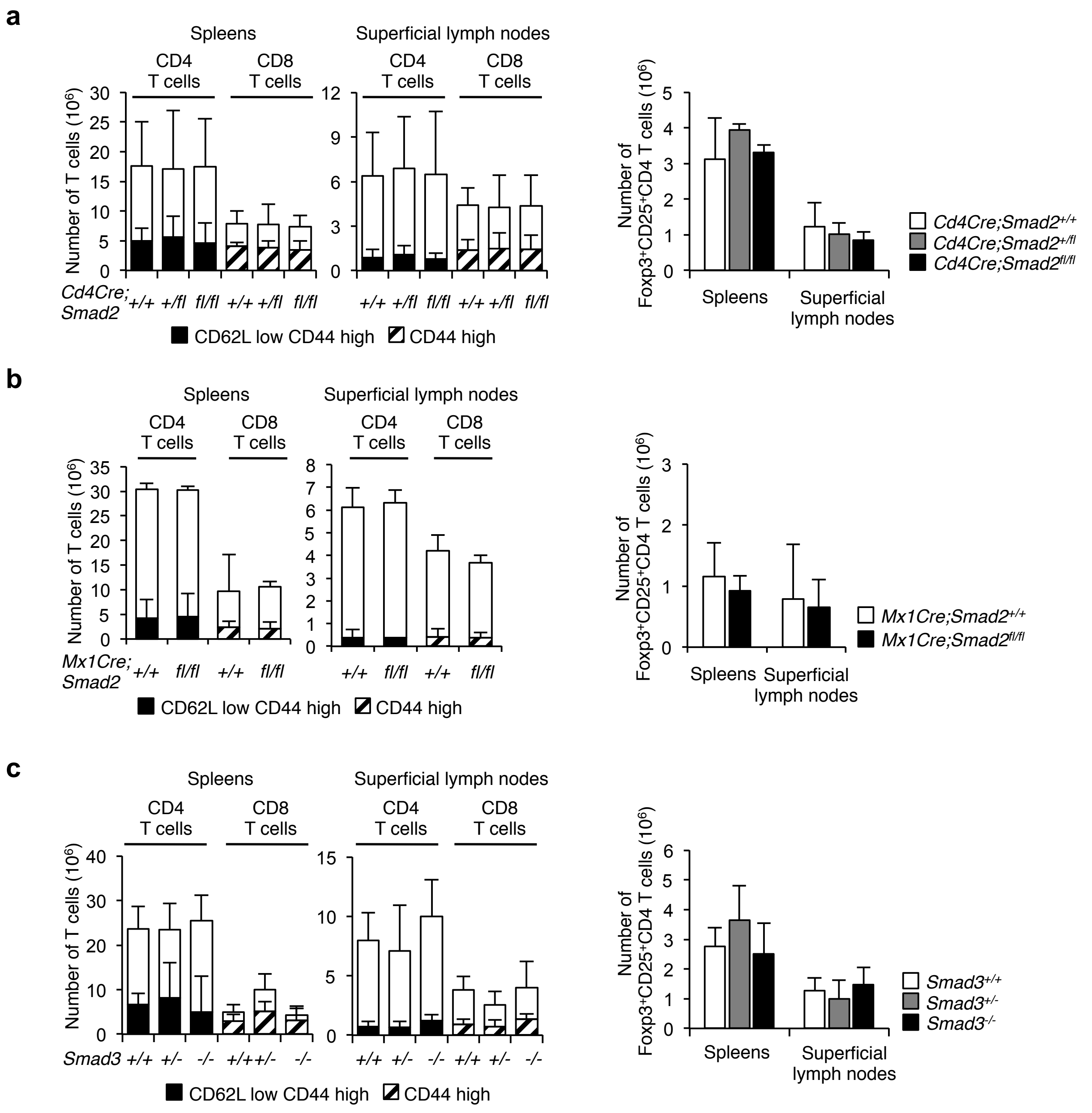

Supplementary Figure 1 Smad2 and Smad3 are not essential for T cell homeostasis. Numbers of naïve/memory CD4⁺ T cells, naïve/memory CD8⁺ T cells, and Foxp3⁺ Tregs in spleens and superficial lymphnodes from (**a**) *Cd4Cre;Smad2*$^{+/+,\ +/fl,\ fl/fl}$ mice, (**b**) *Mx-1Cre;Smad2*$^{+/+,\ fl/fl}$ mice, and (**c**) *Smad3*$^{+/+,\ +/-,\ -/-}$ mice (12-16 weeks of age, *Cd4Cre;Smad2* and *Smad3*, *n* = 10/genotype, *Mx-1Cre;Smad2*, *n* = 5 genotype). Data are from one experiment representative of seven independent experiments. Data are mean + s.d.

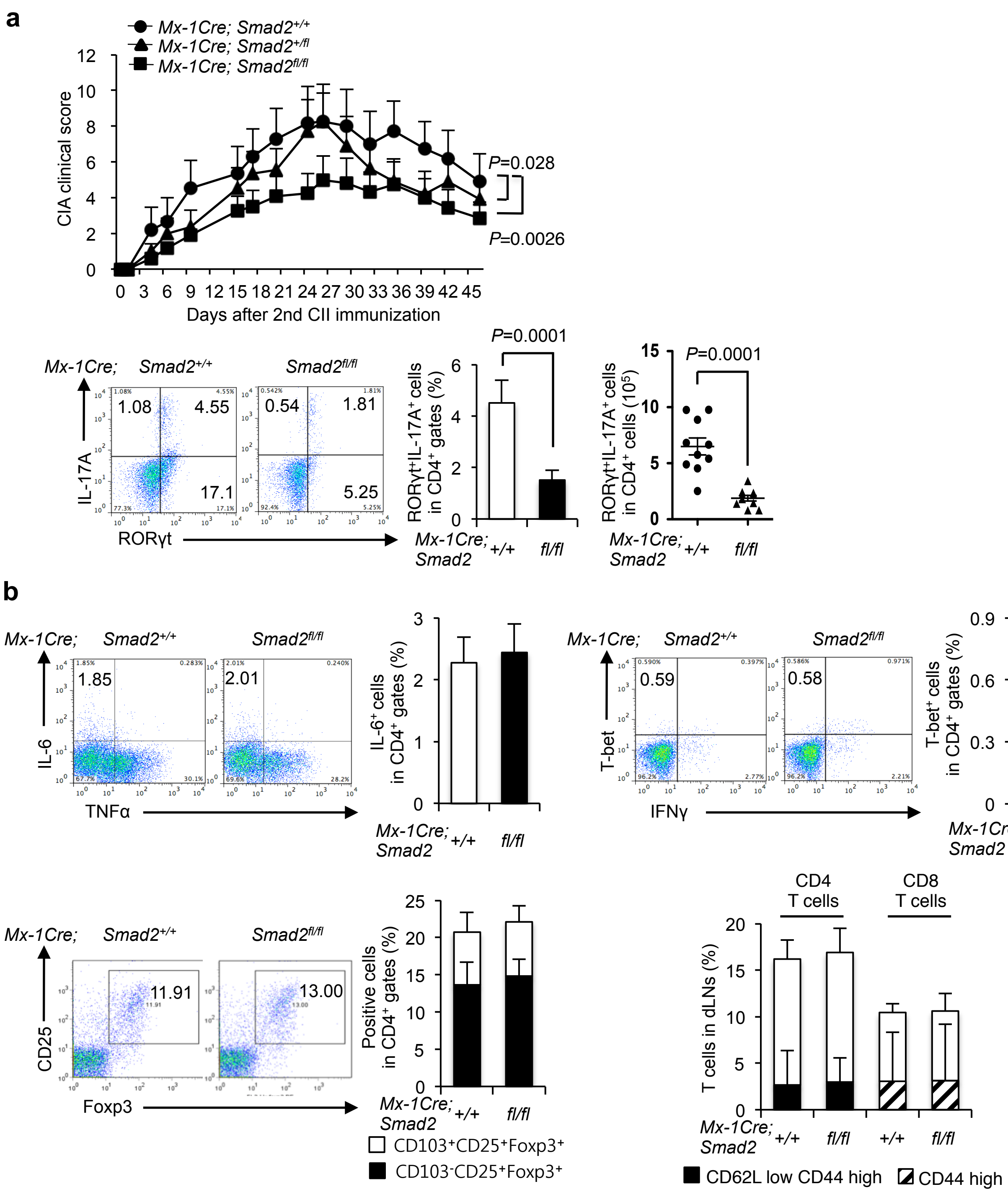

Supplementary Figure 2 Inducible systemic disruption of the *Smad2* gene ameliorates CIA. (a) CIA scoring courses of *Mx-1Cre;Smad2*$^{+/+,\ +/fl,\ fl/fl}$ mice (n = 10/genotype) are shown. Dot plots and graphs of the proportions and numbers of IL-17A^{+}RORγt^{+}CD4^{+} draining lymph node cells from *Mx-1Cre;Smad2*$^{+/+,\ fl/fl}$ mice are shown. (**b**) Dot plots and proportions of IL-6^{+}TNF-α^{+}CD4^{+}, T-bet^{+} IFN-γ^{+}CD4^{+}, CD25^{+}Foxp3^{+}CD4^{+}, CD62L^{high}CD44^{low}, CD62L^{low}CD44^{high} CD4^{+} T cells and CD44^{low}, CD44^{high} CD8^{+} T cells in the draining lymph nodes from *Mx-1Cre;Smad2*$^{+/+,\ fl/fl}$ mice are shown. Data are from one experiment representative of two independent experiments. Data are mean + s.d. with *P* values (two-way ANOVA test).

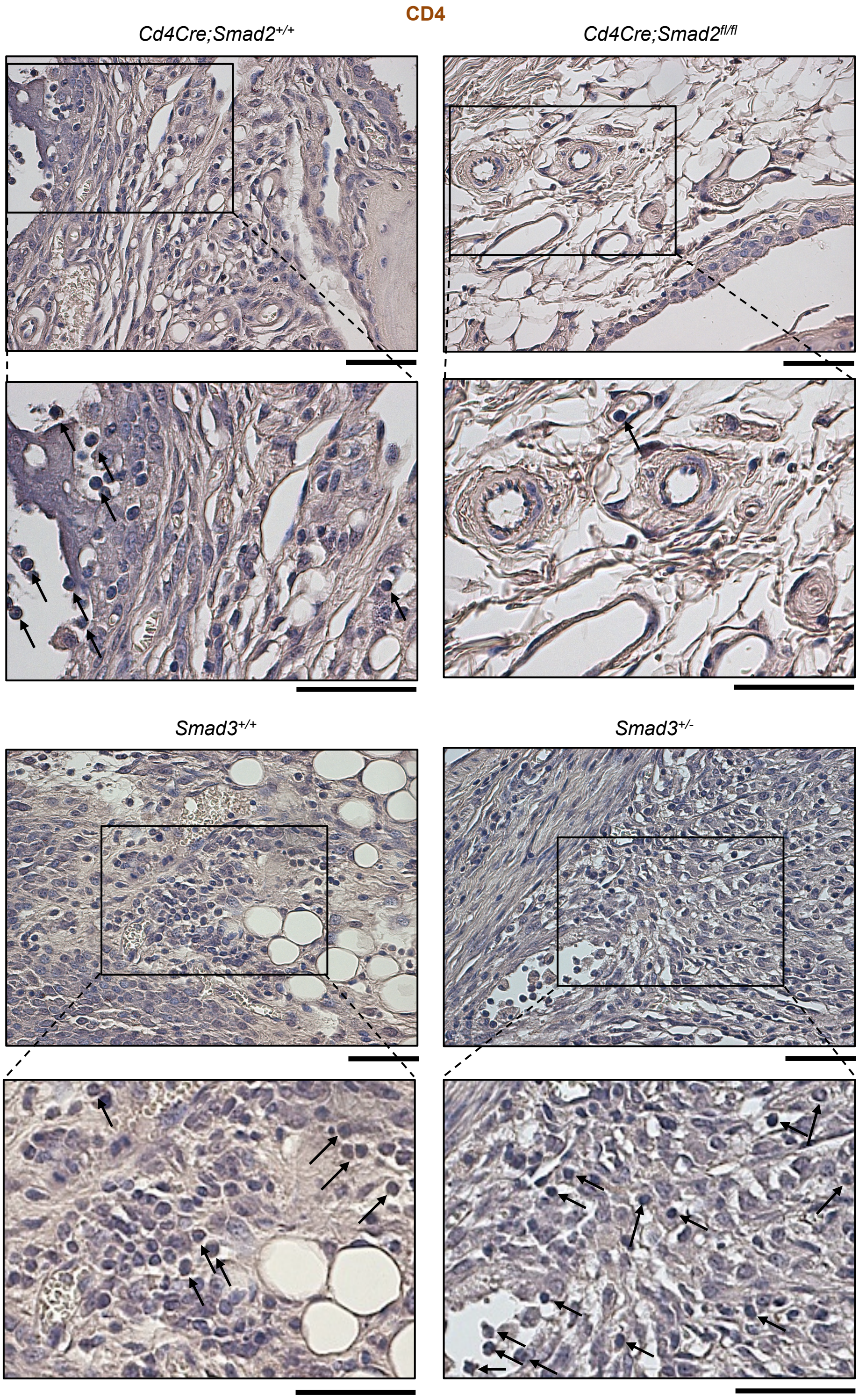

Supplementary Figure 3 Accumulation of CD4$^+$ cells in the joint lesions was ameliorated in *Cd4Cre;Smad2$^{fl/fl}$* mice, whereas it was exacerbated in *Smad3$^{+/-}$* mice. Immunohistochemistry staining of CD4$^+$ in the joint sections (magnification, ×400, scale bars: 50 μm). Data are from one experiment representative of two independent experiments (*Cd4Cre;Smad2* and *Smad3*, *n* = 10/ genotype).

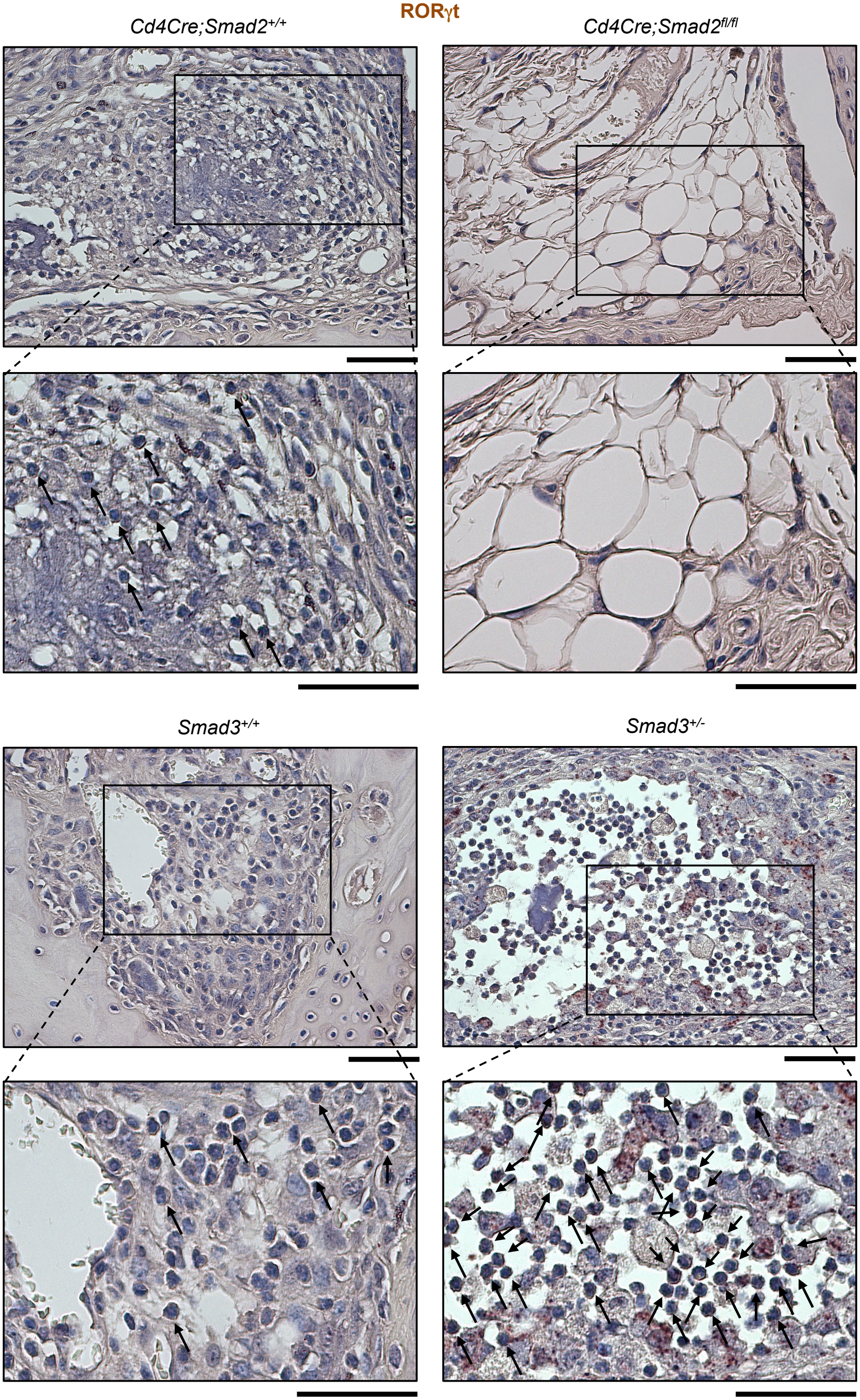

Supplementary Figure 4 Accumulation of RORγt⁺ cells in the joint lesions was ameliorated in *Cd4Cre;Smad2$^{fl/fl}$* mice, whereas it was exacerbated in *Smad3$^{+/-}$* mice. Immunohistochemistry staining of RORγt⁺ in the joint sections (magnification, ×400, scale bars: 50 μm). Data are from one experiment representative of two independent experiments (*Cd4Cre;Smad2* and *Smad3*, *n* = 10/ genotype).

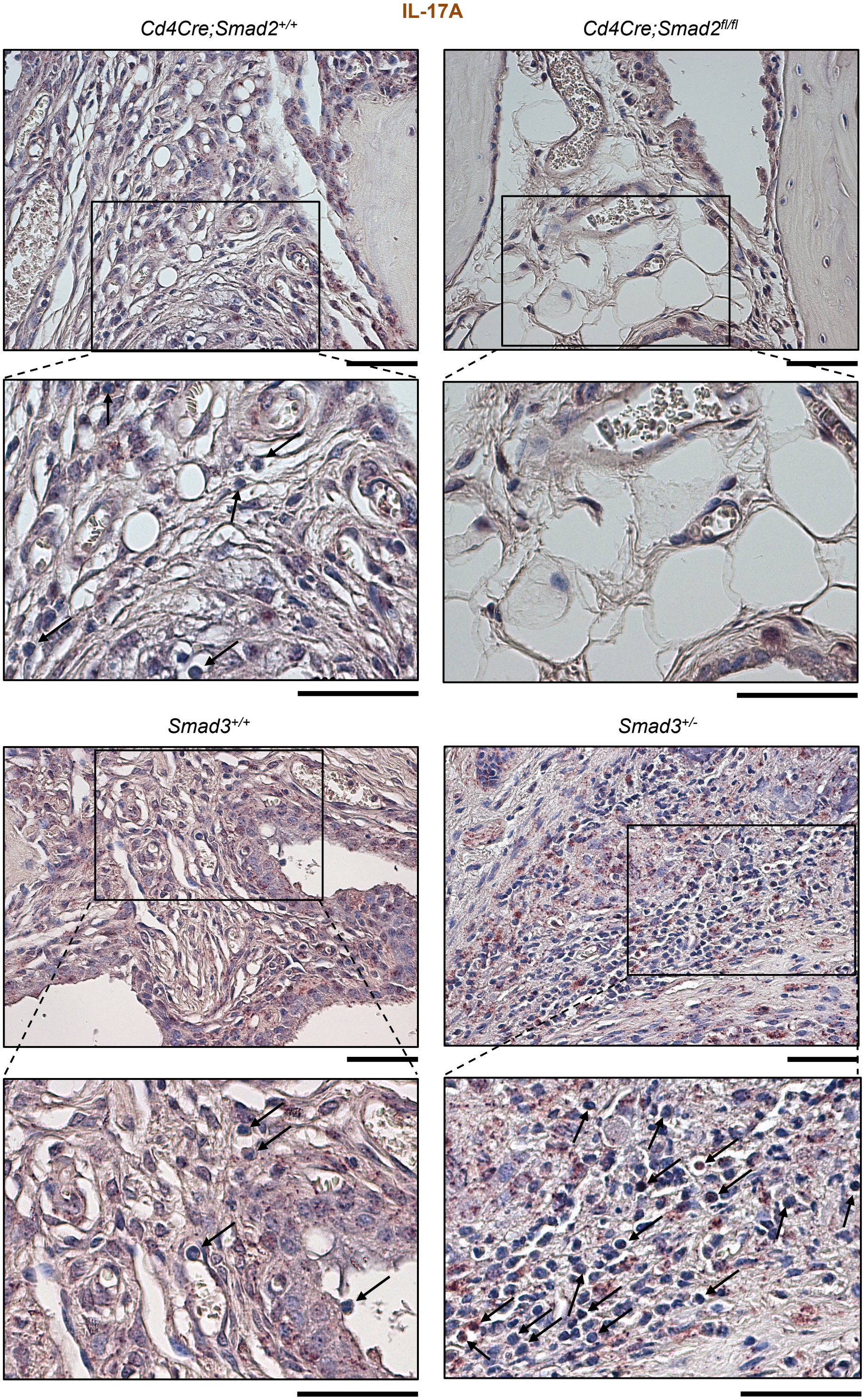

Supplementary Figure 5 Accumulation of IL-17A^{+} cells in the joint lesions was ameliorated in *Cd4Cre;Smad2$^{fl/fl}$* mice, whereas it was exacerbated in *Smad3$^{+/-}$* mice. Immunohistochemistry staining of IL-17A^{+} in the joint sections (magnification, ×400, scale bars: 50 μm). Data are from one experiment representative of two independent experiments (*Cd4Cre;Smad2* and *Smad3*, *n* = 10/ genotype).

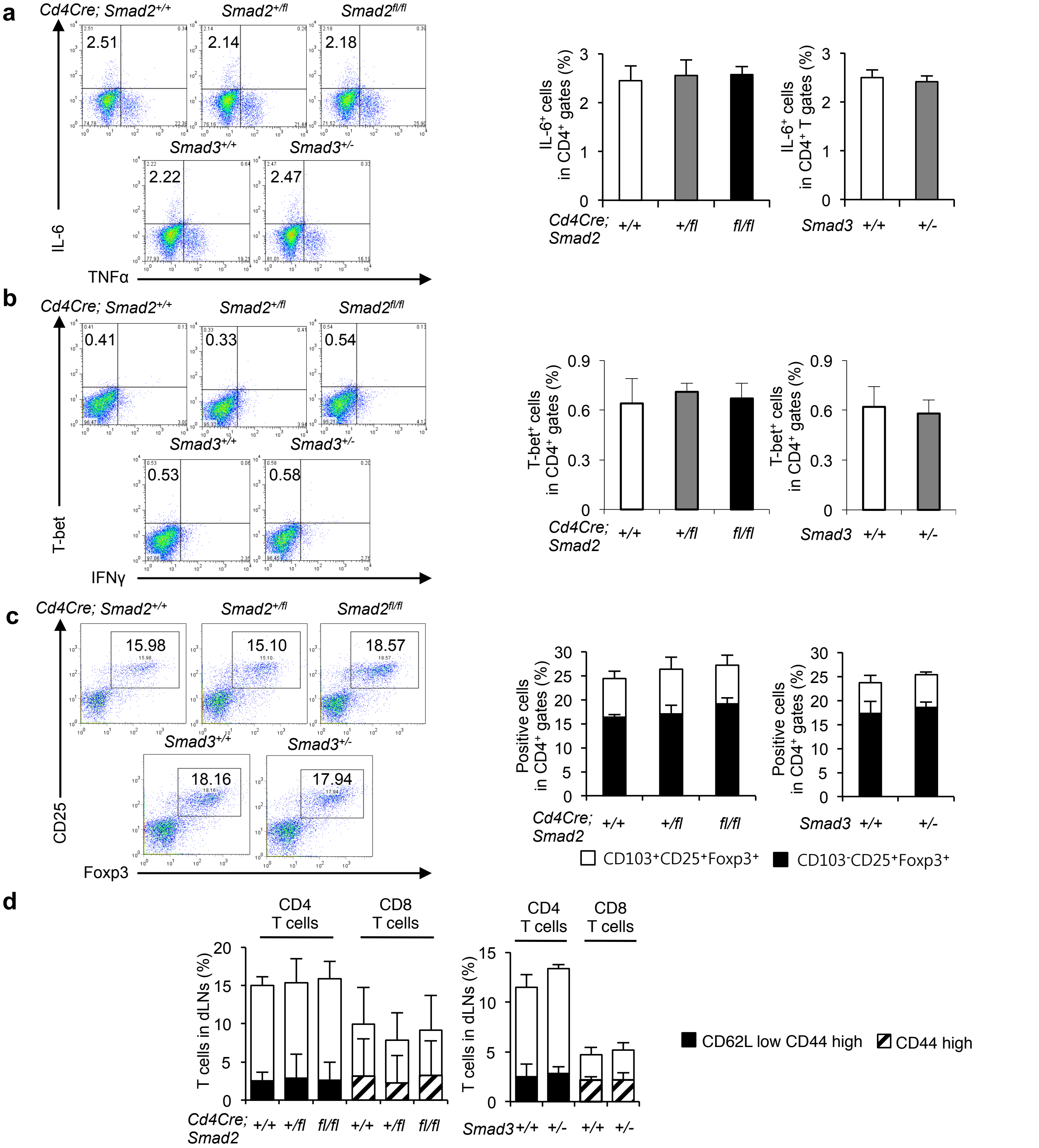

Supplementary Figure 6 Disruption of Smad2 or Smad3 has no effect on effector T cell subsets exceptT_H17 in CIA. Flow cytometry analyses of T cells in the draining lymph nodes of the arthritic joints from *Cd4Cre;Smad2*$^{+/+,\ +/fl,\ fl/fl}$ mice and *Smad3*$^{+/+,\ +/-}$ mice (**a**) IL-6$^+$TNF-α$^+$ in CD4$^+$ gates, (**b**) T-bet$^+$IFN-γ$^+$ in CD4$^+$ gates, (**c**) CD25$^+$Foxp3$^+$ in CD4$^+$ gates, CD103$^-$CD25$^+$Foxp3$^+$ in CD4$^+$ gates, CD103$^+$CD25$^+$Foxp3$^+$ in CD4$^+$ gates, (**d**) CD62L^{high}CD44^{low}, CD62L^{low}CD44^{high} CD4$^+$ T cells and CD44^{low}, CD44^{high} CD8$^+$ T cells (n = 10/genotype). Data are from one experiment representative of four (*Cd4Cre;Smad2*$^{+/+,\ fl/fl}$, n = 20/genotype, *Cd4Cre;Smad*$^{+/fl}$, n = 15) or three (*Smad3*, n = 15/genotype) independent experiments. Data are mean + s.d.

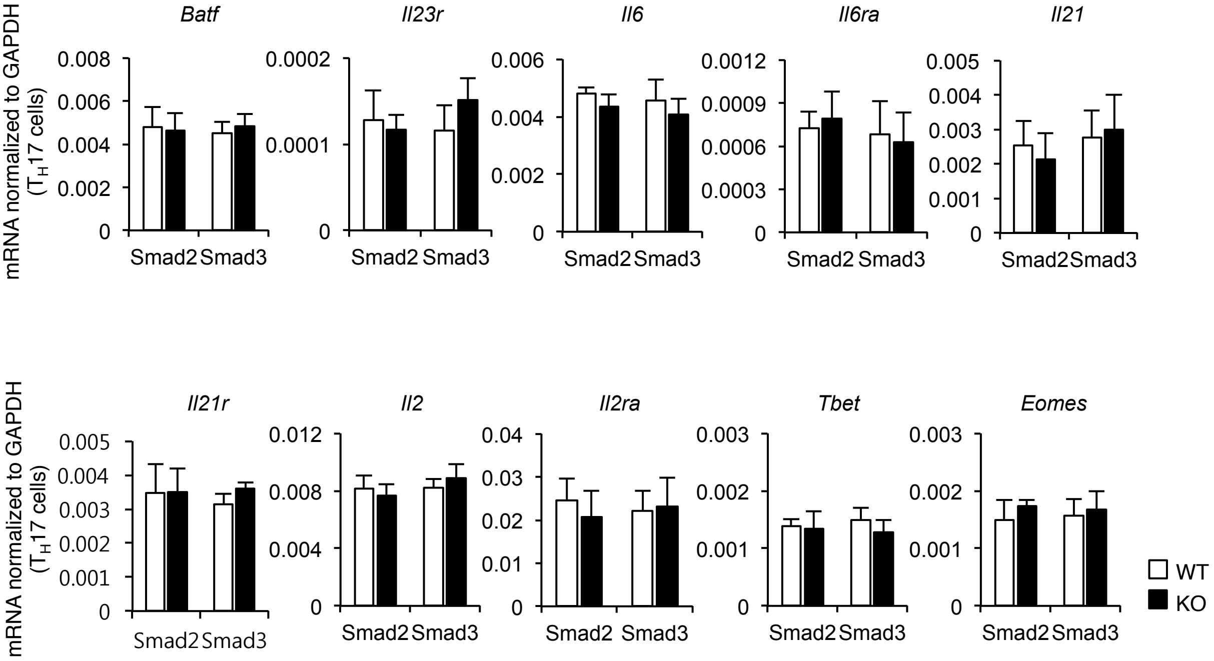

Supplementary Figure 7 Disruption of Smad2 or Smad3 has no effect on T_H17-related genes except the *Rorc* and *Il17a* genes. Quantitative RT-PCR analysis of *Batf*, *Il23r*, *Il6*, *Il6ra*, *Il21*, *Il21r*, *Il2*, *Il2ra*, *Tbet*, *Eomes* to *Gapdh* in *Smad2*$^{+/+,\ -/-}$ and *Smad3*$^{+/+,\ -/-}$ T_H17 cells. Data are pooled from three independent experiments. Each experiment was performed in triplicate (*n* = 3). Data are mean + s.d.

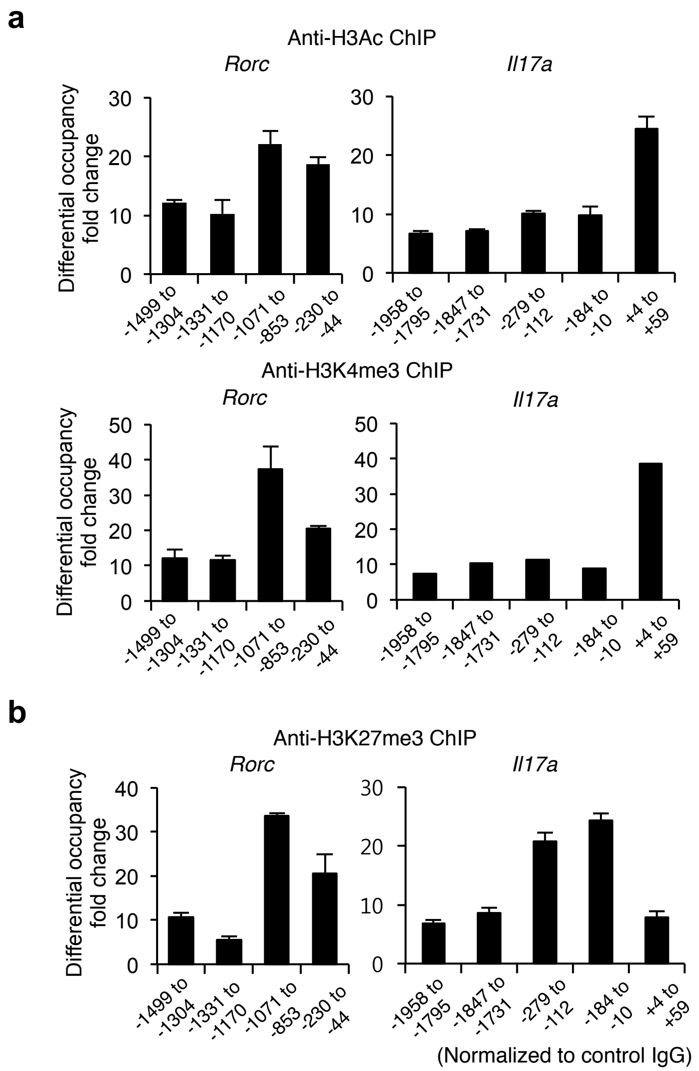

Supplementary Figure 8 Smad2 binding sites are associated with active epigenetic marks, whereas Smad3 binding sites are associated with inactive epigenetic marks in the *Rorc* and *Il17a* promoter regions. ChIP analysis of T_H17 cells with antibodies against (**a**) acetylated histone H3 (H3Ac), trimethylated histone H3 Lys4 (H3K4me3), and (**b**) trimethylated histone H3 Lys 27 (H3K27me3). Data are from one experiment representative of two independent experiments. Each experiment was performed in triplicate (*n* = 3). Data are mean + s.d.

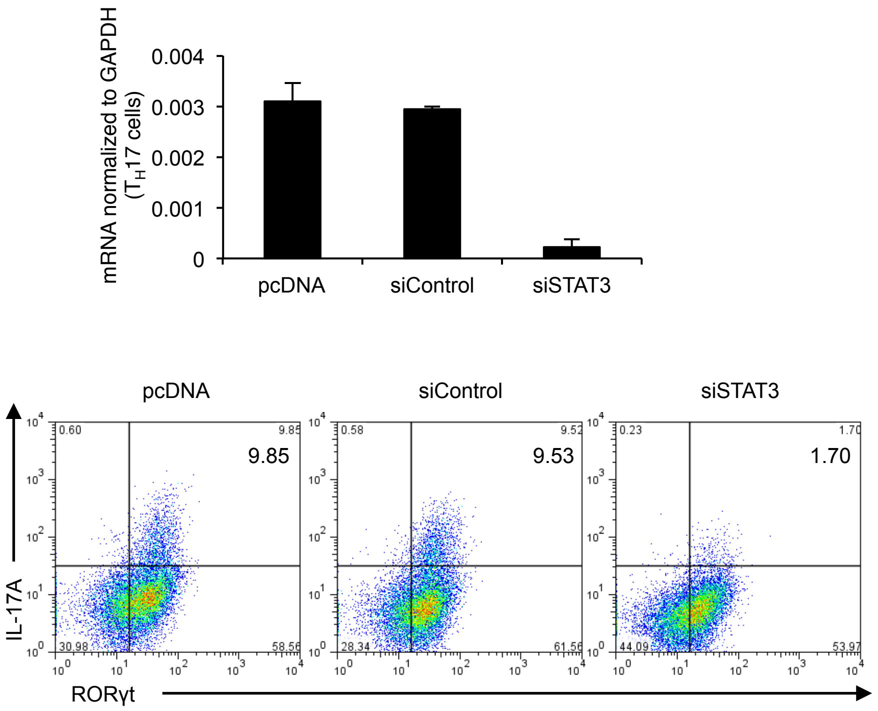

Supplementary Figure 9 Knockdown of the *Stat3* gene using STAT3 siRNA in T_H17 cells. Stat3 mRNA levels in T_H17 cells transduced with the controls or STAT3 siRNA were confirmed by quantitative RT-PCR. Flow cytometry analyses of IL-17A$^+$RORγt$^+$ in CD4$^+$ gates transduced with pcDNA, control siRNA or STAT3 siRNA in T_H17-polarizing condition. Data are from one experiment representative of two independent experiments. Each experiment was performed in triplicate (n = 3). Data are mean + s.d.

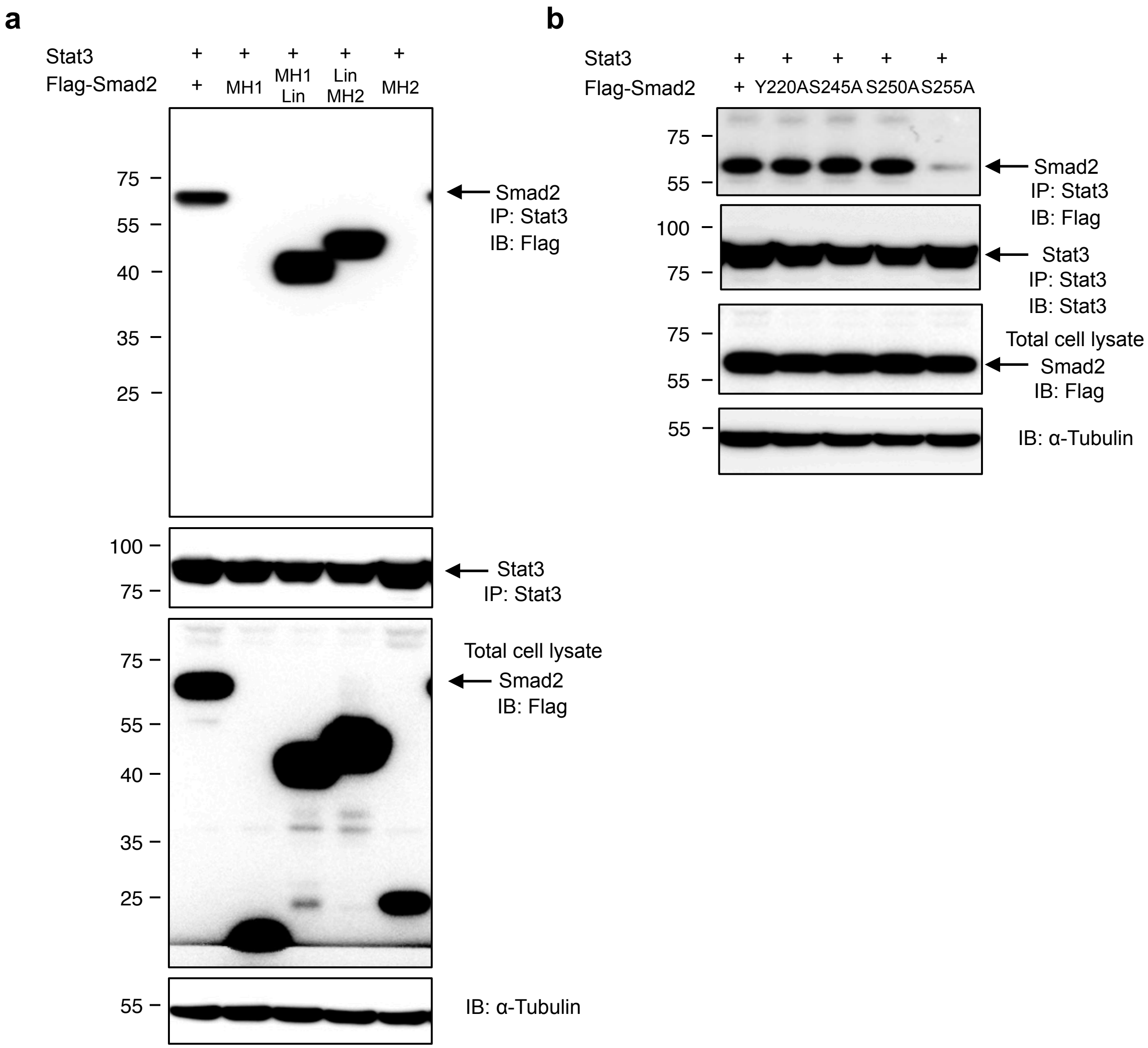

Supplementary Figure 10 Linker-phosphorylated Smad2 at S255 interacts with STAT3. Interactions of exogenous Smad2 proteins and STAT3 protein in 293T cells were determined by immunoprecipitation. (**a**) Effects of truncated mutations in Smad2 on the interaction with STAT3 in 293T cells. (**b**) Effects of linker domain variations in Smad2 on the interaction with STAT3 in 293T cells. Data are from one experiment representative of three independent experiments.

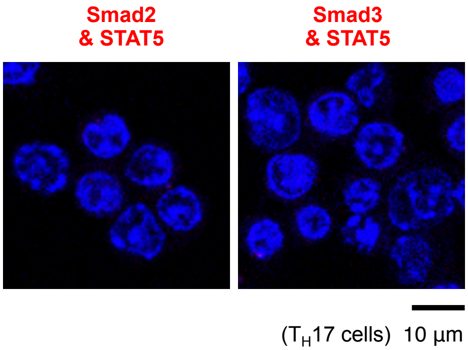

Supplementary Figure 11 TGF-β R-Smads do not interact with STAT5 in T_H17 cells. PLA shows no close proximity between Smad2/3 and STAT5 in T_H17 cells (scale bar: 10 μm). Data are from one experiment representative of three independent experiments.

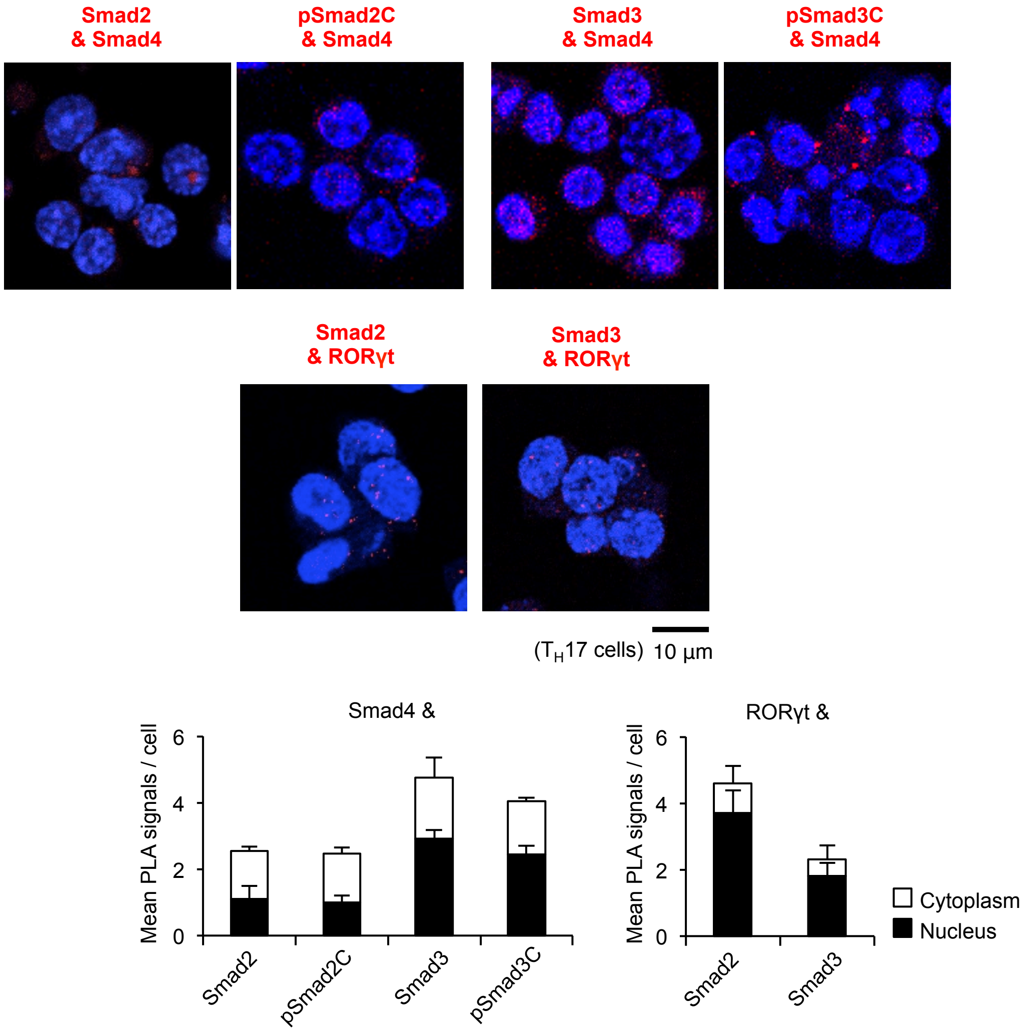

Supplementary Figure 12 TGF-β R-Smads show close proximity with Smad4 or RORγt in T_H17 cells. PLA shows the endogenous close proximity between Smad2-Smad4, pSmad2C-Smad4, Smad3-Smad4, pSmad3C-Smad4, Smad2-RORγt, and Smad3-RORγt in T_H17 cells. PLA signals were quantified using BlobFinder software (scale bars: 10 μm, nucleus: black, cytoplasm: white, *n* = 10 fields). Data are from one experiment representative of three independent experiments. Data are mean + s.d.

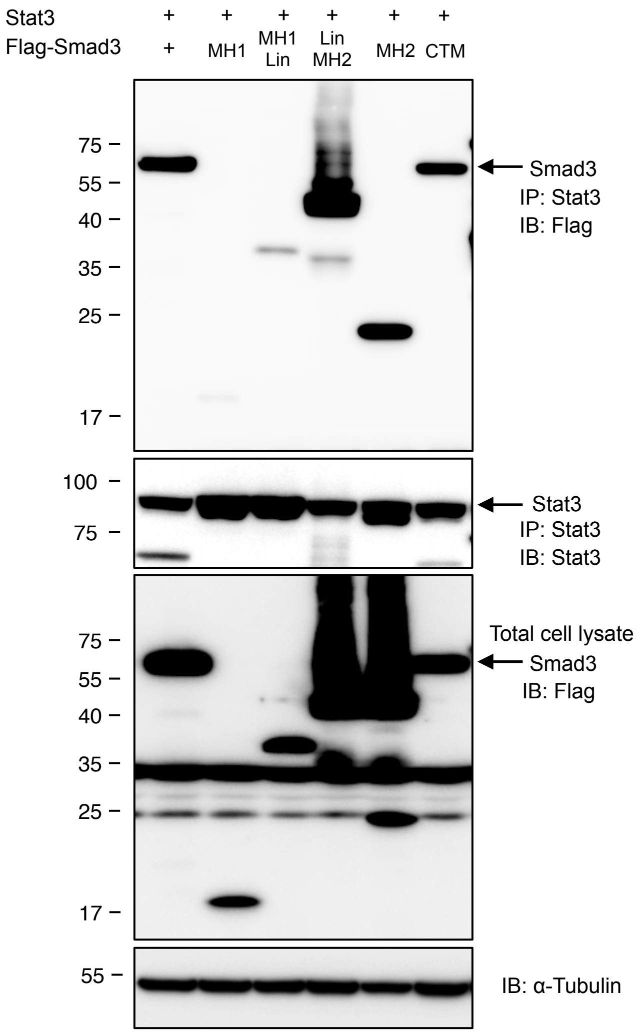

Supplementary Figure 13 Smad3 MH2 domain interacts with STAT3. Interactions of Smad3 or various Smad3 mutants and STAT3 in 293T cells were determined by immunoprecipitation. Data are from one experiment representative of three independent experiments.

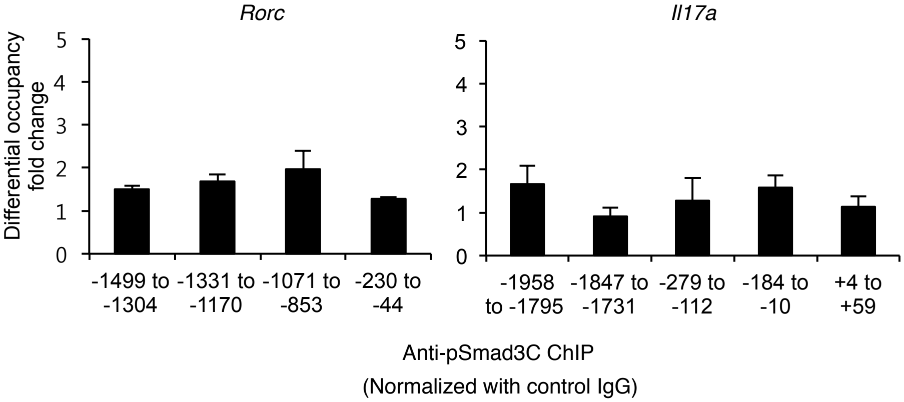

Supplementary Figure 14 C-terminally phosphorylated Smad3 does not bind to the PIAS3/ Smad3-binding sites in the *Rorc* and *Il17a* proximal promoter regions in T_H17 cells. ChIP with the antibody against pSmad3C shows that pSmad3C does not bind to the PIAS3/Smad3-binding sites in the *Rorc* and *Il17a* proximal promoter regions of T_H17 cells. Data are from one experiment representative of two independent experiments. Each experiment was performed in triplicate (n = 3). Data are mean + s.d.

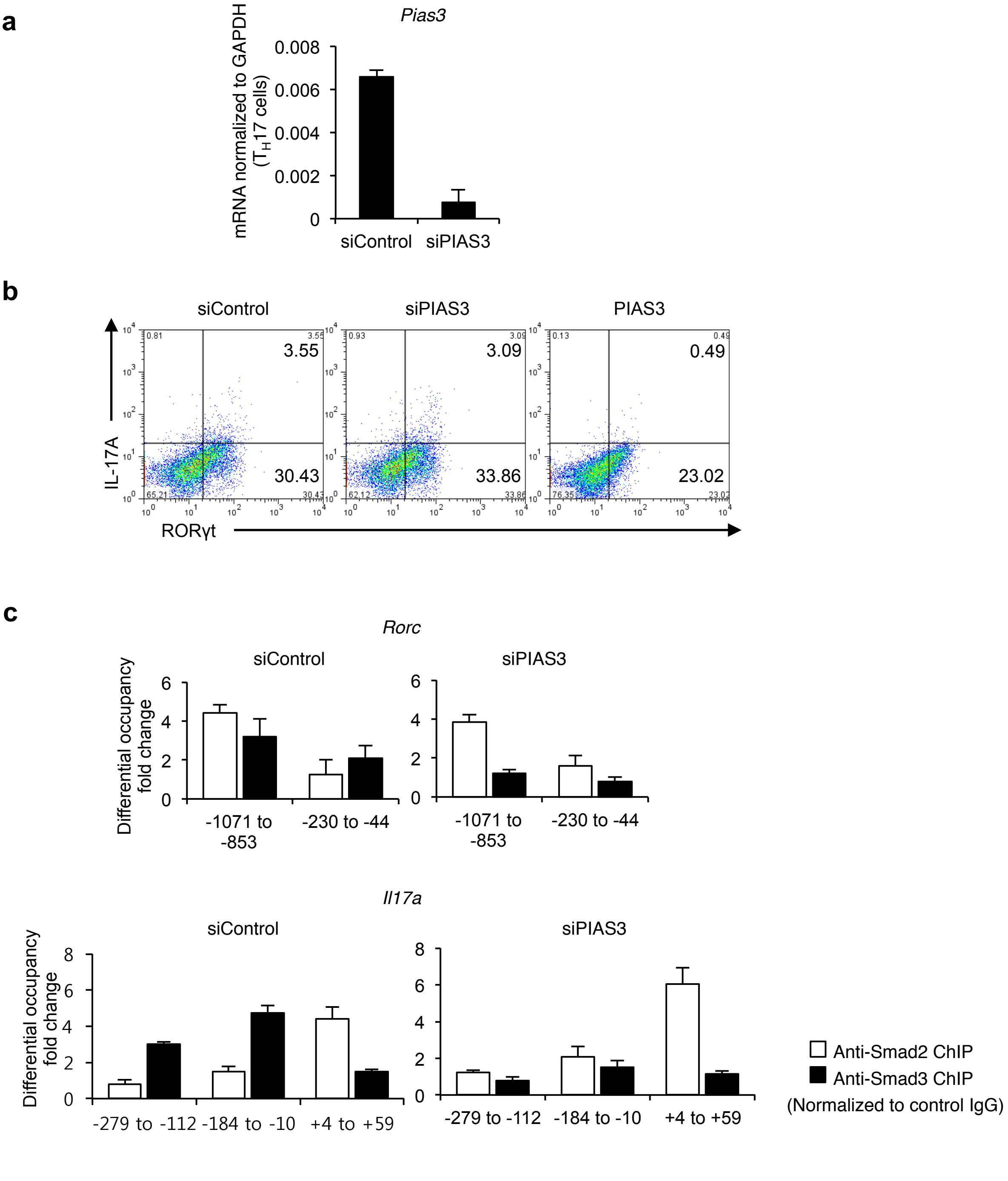

Supplementary Figure 15 PIAS3 is required for Smad3 to bind to the *Rorc* and the *Il17a* promoter regions. (**a**) PIAS3 mRNA levels in T_H17 cells transduced with the control siRNA or PIAS3 siRNA were confirmed by quantitative RT-PCR. (**b**) Flow cytometry analyses of IL-17A$^+$ RORγt$^+$CD4$^+$ T cells transduced with control siRNA, PIAS3 siRNA or PIAS3 in T_H17-polarizing condition. (**c**) ChIP analysis of T_H17 cells transduced with control siRNA or PIAS3 siRNA with the antibodies against Smad2 and Smad3. One experiment was performed in triplicate (n = 3). Data are mean + s.d.

a

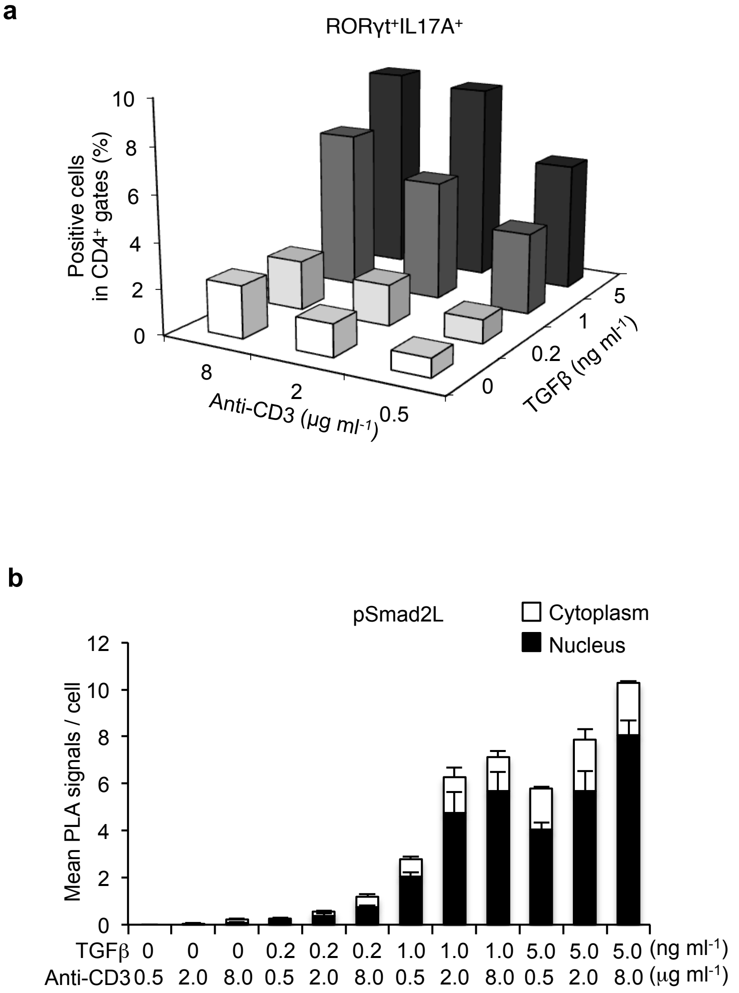

Supplementary Figure 16 Strength of TGF-β and TCR signals correlate with T_H17 differentiation and pSmad2L. Purified $CD4^+$ T cells were activated under T_H17-polarizing condition with the indicated doses of TGF-β and plate-coated anti-CD3 for 3 days. (**a**) Percentages of IL-17A$^+$RORγt$^+$CD4$^+$ T cells determined by flow cytometry. (**b**) Expression of pSmad2L in $CD4^+$ T cells determined by PLA. PLA signals were quantified using BlobFinder software (nucleus: black, cytoplasm: white, n = 10 fields). Data are from one experiment representative of two independent experiments. Data are mean + s.d.

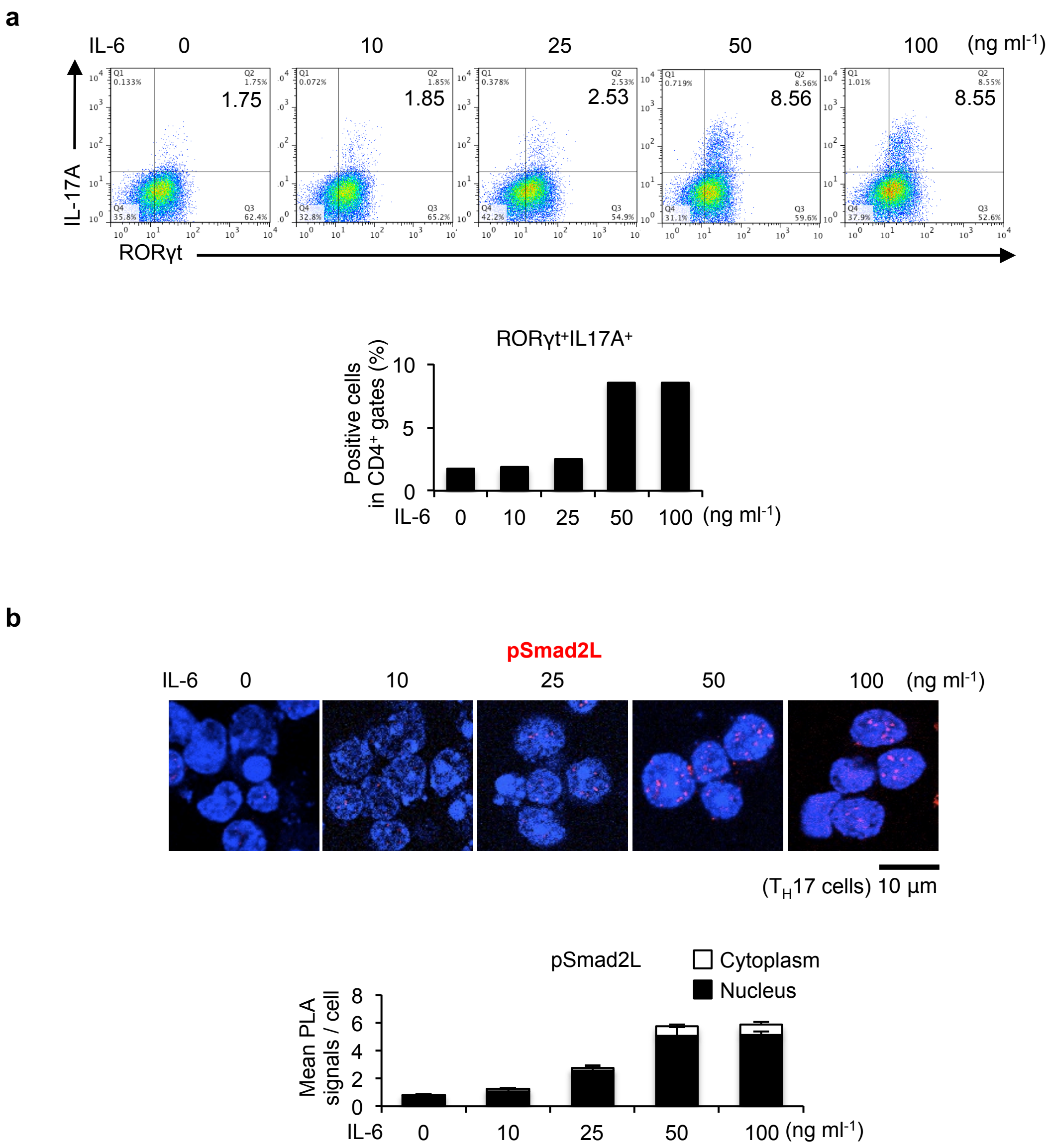

Supplementary Figure 17 IL-6 doses correlate with T_H17 differentiation. Purified CD4$^+$ T cells were activated under T_H17-polarizing condition with the indicated doses of IL-6 for 3 days. (**a**) Flow cytometry analyses of IL-17A$^+$RORγt$^+$CD4$^+$ T cells. (**b**) Expression of pSmad2L in CD4$^+$ T cells determined by PLA. PLA signals were quantified using BlobFinder software (scale bars: 10 μm, nucleus: black, cytoplasm: white, *n* = 10 fields). Data are from one experiment representative of two independent experiments. Data are mean + s.d.

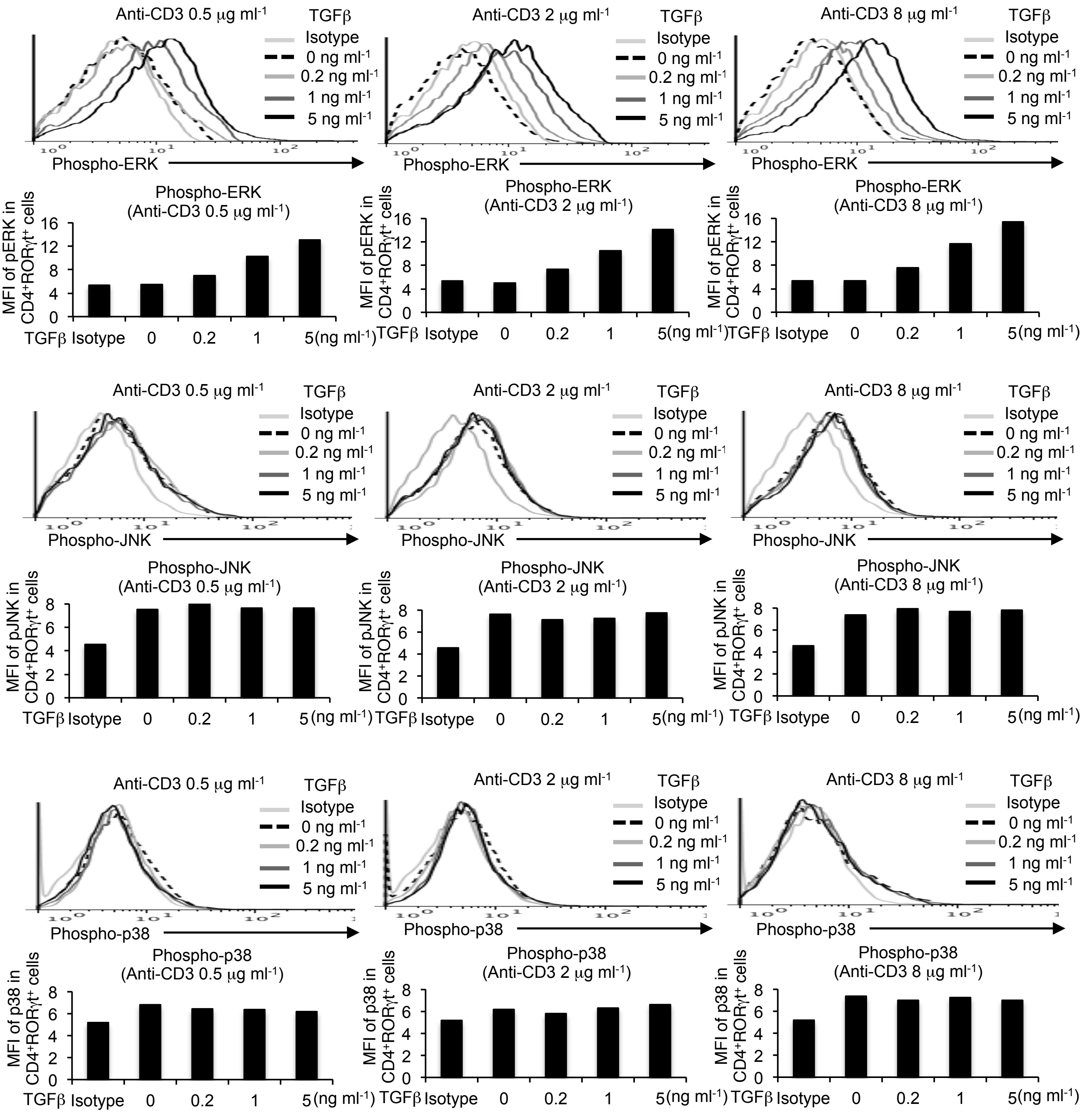

Supplementary Figure 18 Strength of TGF-β and TCR signals correlate with ERK phosphorylation . Purified $CD4^+$ T cells were activated under T_H17-polarizing condition with the indicated doses of TGF-β ,and plate-coated anti-CD3 for 3 days. Flow cytometry analyses of phospho-ERK, phospho-JNK, and phospho-p38 in $ROR\gamma t^+CD4^+$ gate. Graphs show mean fluorescence intensity (MFI). Data are from one experiment representative of two independent experiments.

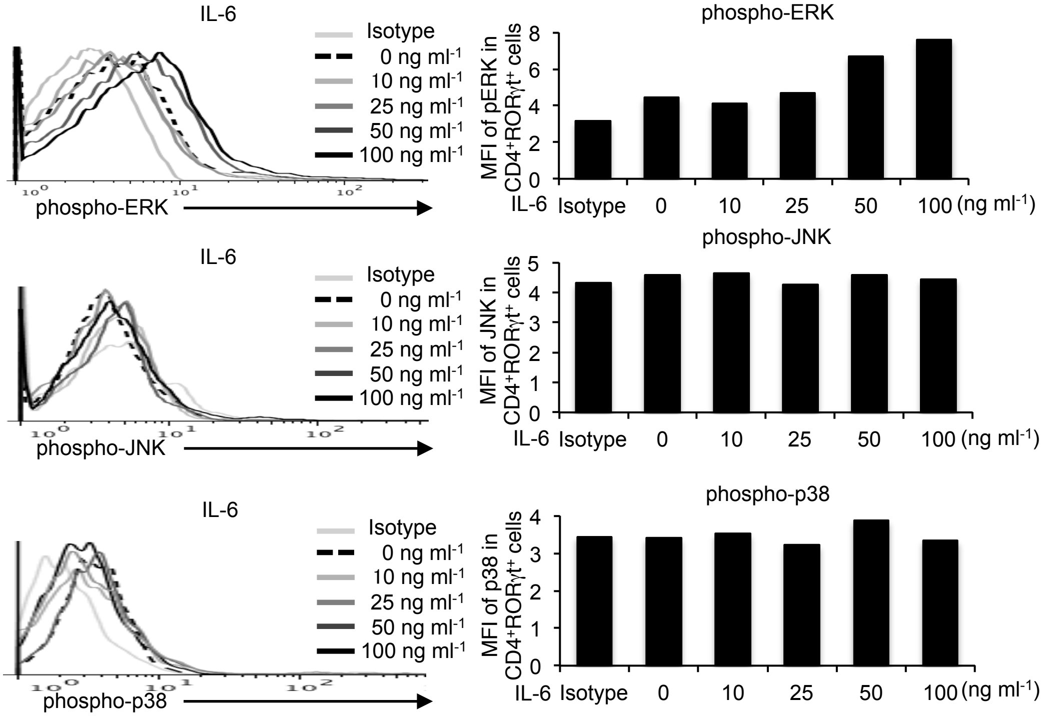

Supplementary Figure 19 High doses IL-6 enhance ERK phosphorylation. Purified CD4$^+$ T cells were activated under T_H17-polarizing condition with the indicated doses of IL-6 for 3 days. Flow cytometry analyses of phospho-ERK, phospho-JNK, and phospho-p38 in RORγt$^+$CD4$^+$ gate. Graphs show mean fluorescence intensity (MFI). Data are from one experiment representative of two independent experiments.

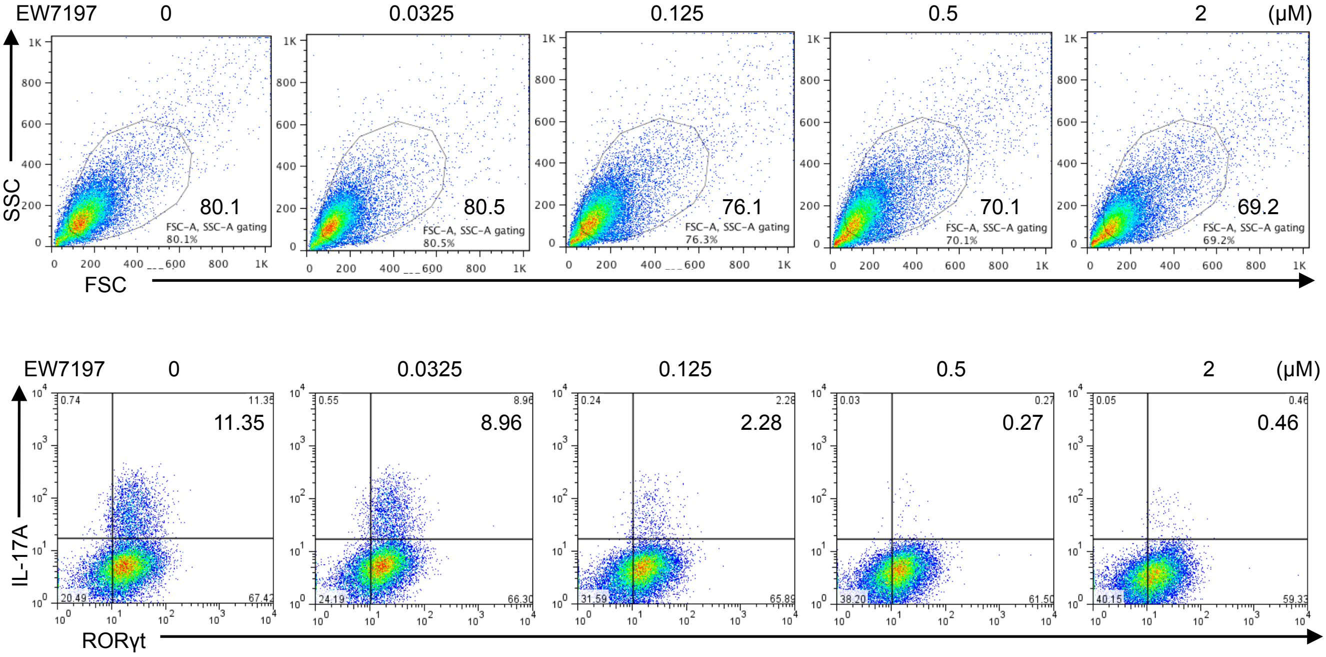

Supplementary Figure 20 Viability and T_H17 differentiation of $CD4^+$ T cells treated with ALK5 inhibitor, EW-7197. Purified $CD4^+$ T cells were activated under T_H17-polarizing condition with the indicated doses of EW-7197 for 3 days. Flow cytometry analyses of FSC/SSC and IL-$17A^+$ $RORγt^+CD4^+$ T cells. Data are from one experiment representative of two independent experiments.

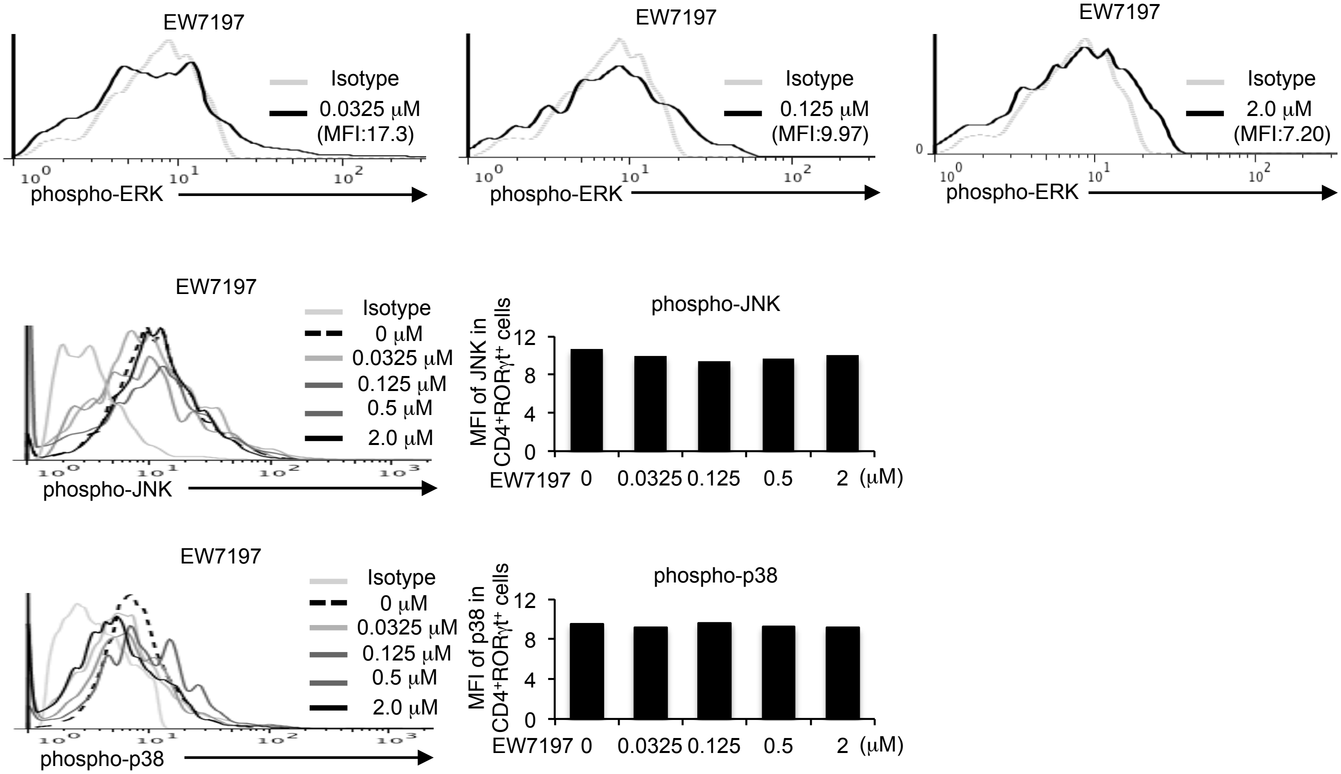

Supplementary Figure 21 ALK5 inhibitor, EW-7197 suppresses ERK phosphorylation, but not phosphorylation of JNK and p38. Purified $CD4^+$ T cells were activated under T_H17-polarizing condition with the indicated doses of an ALK5 inhibitor: EW-7197 for 3 days. Flow cytometry analyses of phospho-ERK, phospho-JNK, and phospho-p38 in $ROR\gamma t^+CD4^+$ gate. Graphs show mean fluorescence intensity (MFI). Data are from one experiment representative of two independent experiments.

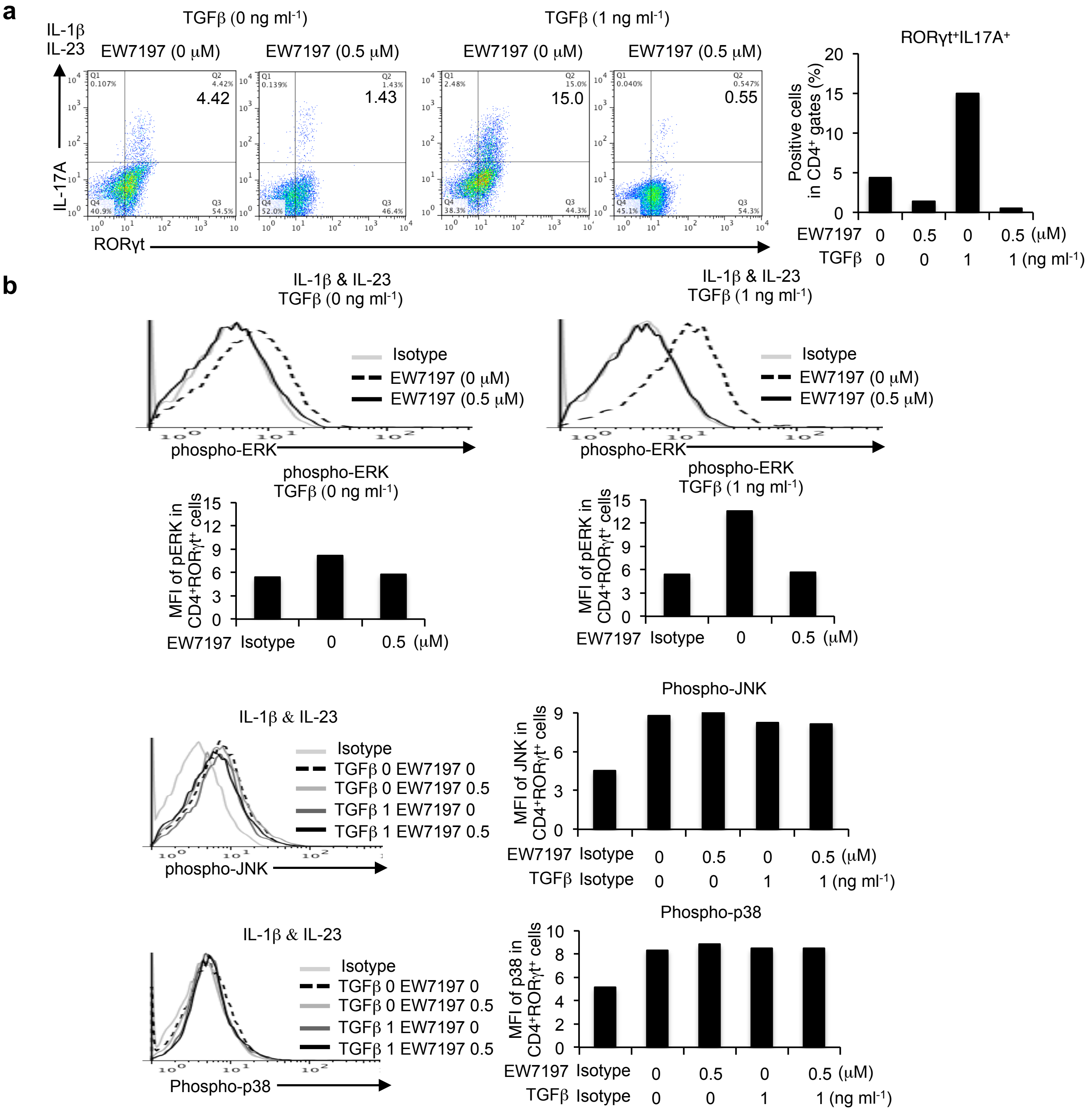

Supplementary Figure 22 TGF-β enhances, whereas EW-7197 suppresses ERK phosphorylation and T_H17 differentiation induced by IL-6, IL-23 and IL-1β. Purified $CD4^+$ T cells were activated under T_H17-polarizing condition with IL-6, IL-23, and IL-1β in the presence or absence of TGF-β1 (1 ng ml^{-1}) or EW-7197 (0.5 μM) for 3 days. (**a**) Flow cytometry analyses of IL-$17A^+$RORγ$t^+$$CD4^+$ T cells. The graph shows the percentages of IL-$17A^+$RORγt^+ cells in $CD4^+$ gates are shown. (**b**) Flow cytometry analyses of phospho-ERK, phospho-JNK, and phospho-p38 in RORγ$t^+$$CD4^+$ gate. Graphs show mean fluorescence intensity (MFI). Data are from one experiment representative of two independent experiments.

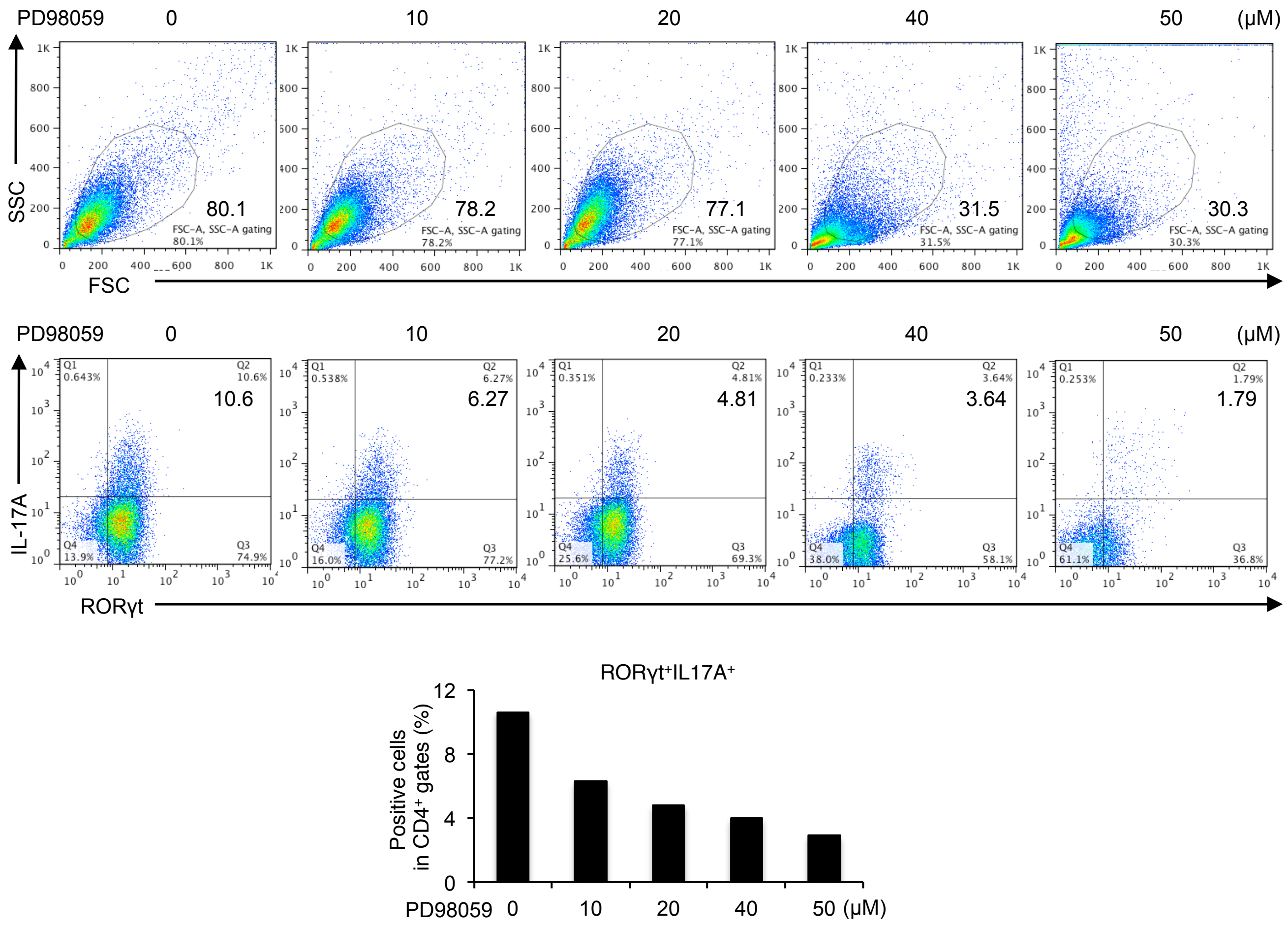

Supplementary Figure 23 Viability and T_H17 differentiation of $CD4^+$ T cells treated with MEK inhibitor, PD98059. Purified $CD4^+$ T cells were activated under T_H17-polarizing condition with the indicated doses of PD98059 for 3 days. Flow cytometry analyses of FSC/SSC and IL-$17A^+$ROR$\gamma t^+$$CD4^+$ T cells. Percentages of positive cell populations are shown. Data are from one experiment representative of two independent experiments.

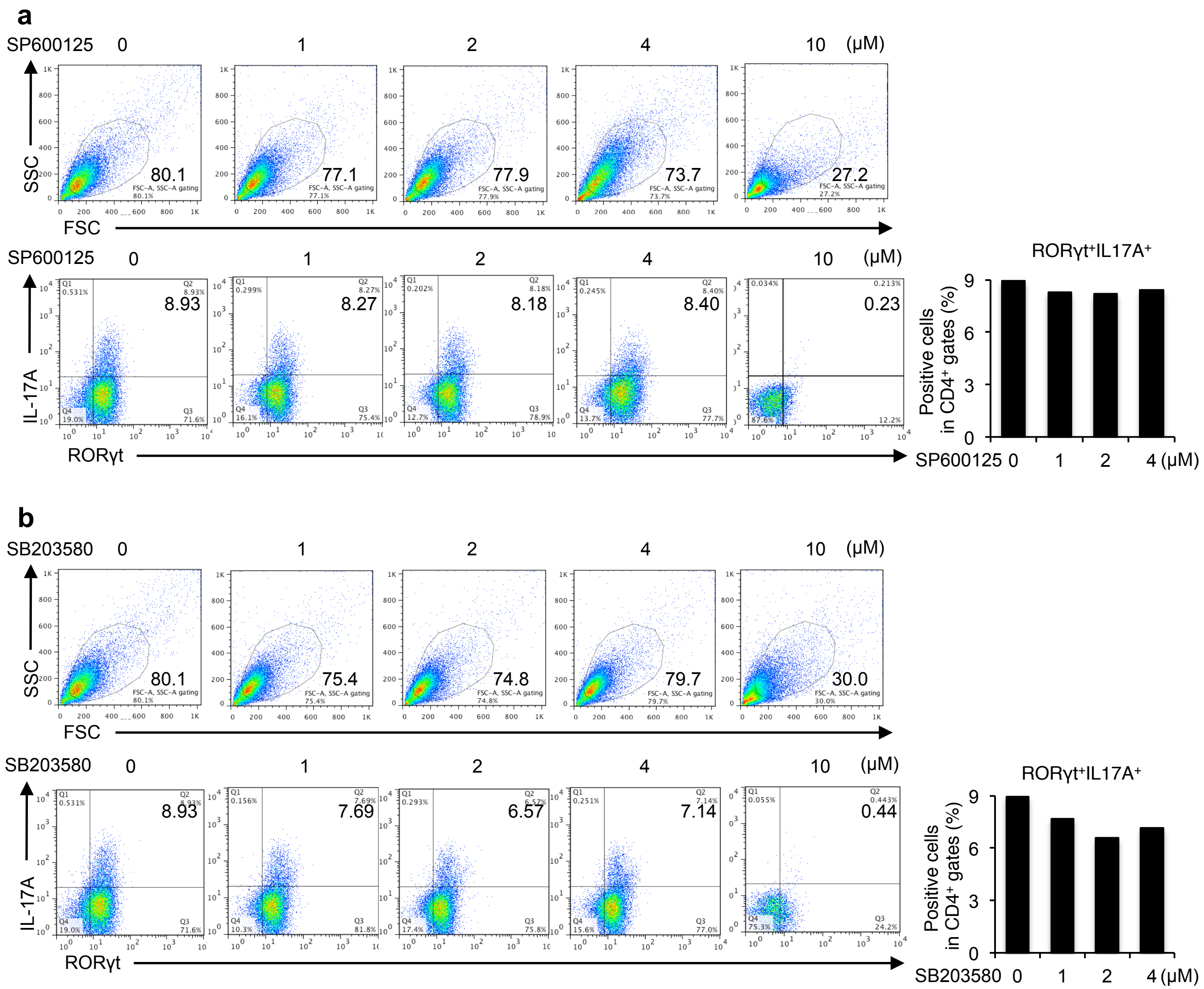

Supplementary Figure 24 Inhibitors of JNK and p38 do not suppress T_H17 differentiation. Purified $CD4^+$ T cells were activated under T_H17-polarizing condition with the indicated doses of (**a**) a JNK inhibitor: SP600125 or (**b**) p38 inhibitor: SB203580 for 3 days. Flow cytometry analyses of FSC/SSCand IL-17A$^+$RORγt$^+$CD4$^+$ T cells. Data are from one experiment representative of two independent experiments.

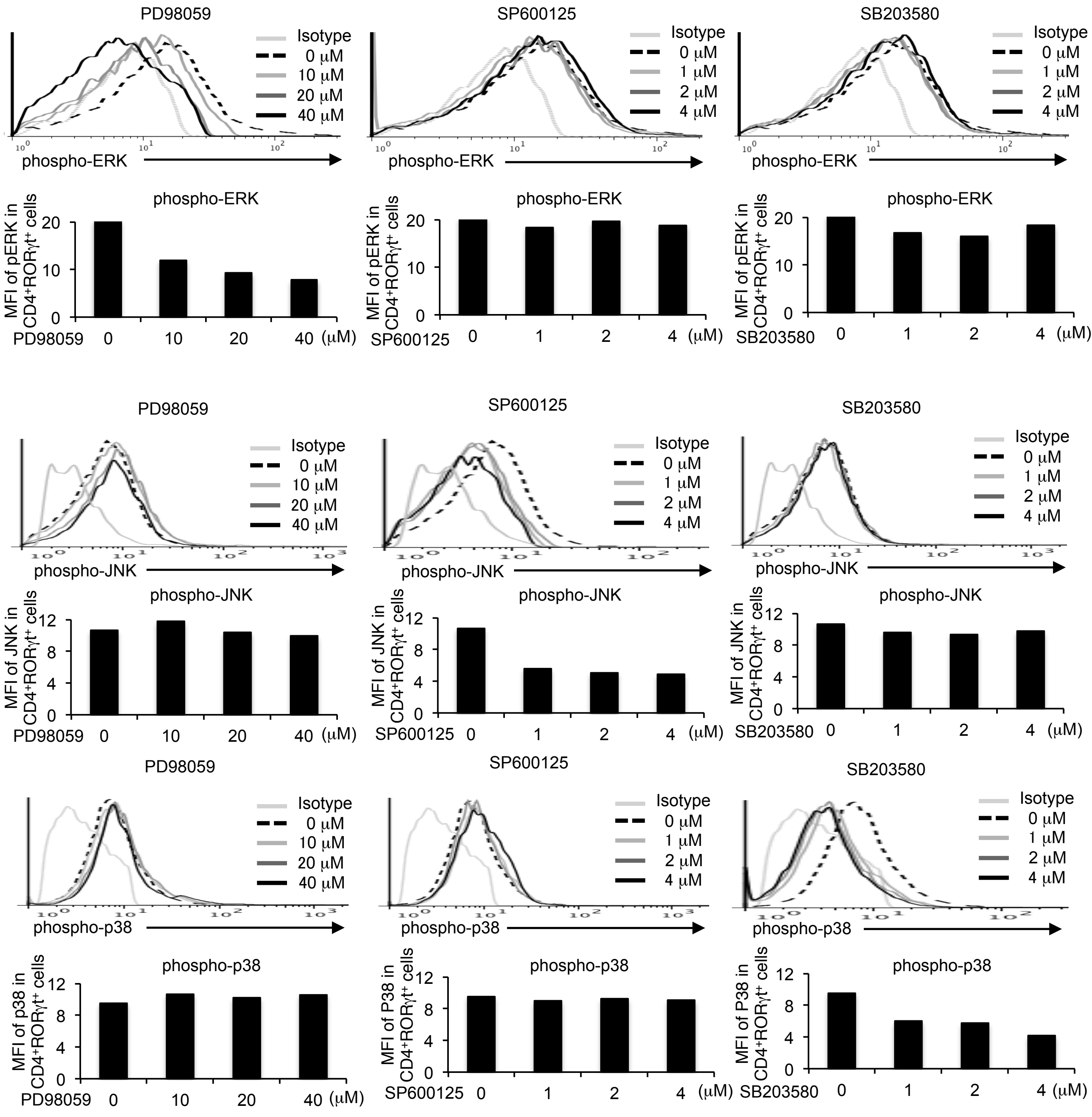

Supplementary Figure 25 Specificity of MAPK inhibitors. Purified $CD4^+$ T cells were activated underT_H17-polarizing condition with the indicated doses of MAPK inhibitors (PD98059, SP600125 , and SB203580) for 3 days. Flow cytometry analyses of phospho-ERK, phospho-JNK, and phospho-p38 in $ROR\gamma t^+CD4^+$ gate. Graphs show mean fluorescence intensity (MFI). Data are from one experiment representative of two independent experiments.

a

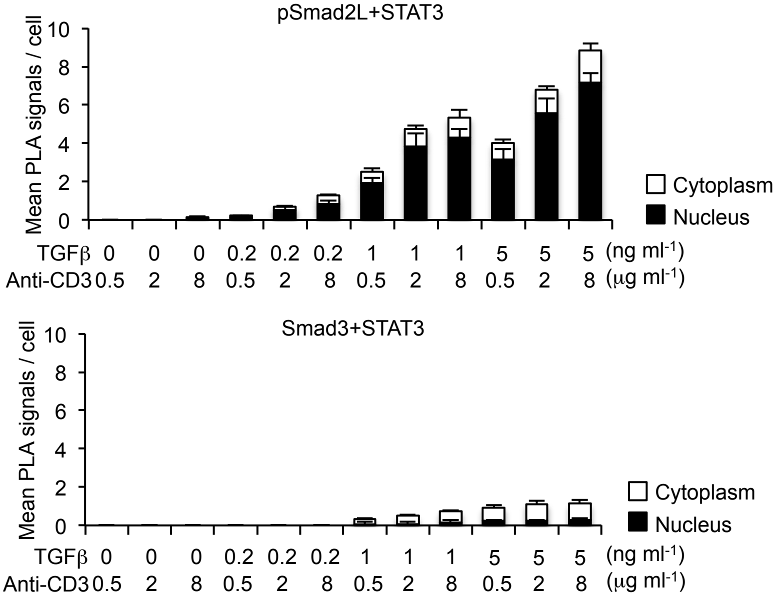

b

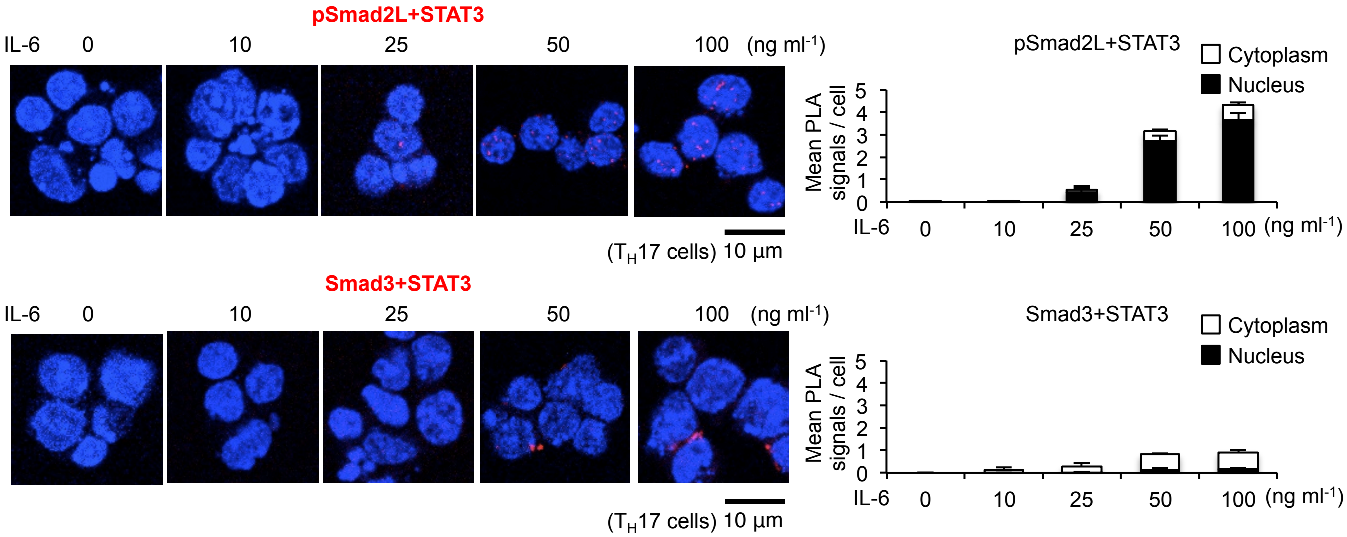

Supplementary Figure 26. Higher doses of (a) TGF-β1, anti-CD3 antibody, and (b) IL-6 upregulated pSmad2L-STAT3 interaction in the nuclei with miniscule changes in Smad3-STAT3 interaction. Expression of pSmad2L-STAT3 and Smad3-STAT3 was quantified by PLA. PLA signals were quantified using BlobFinder software (scale bars: 10 μm, nucleus: black, cytoplasm: white, $n = 10$ fields). Data are from one experiment representative of two independent experiments. Data are mean + s.d.

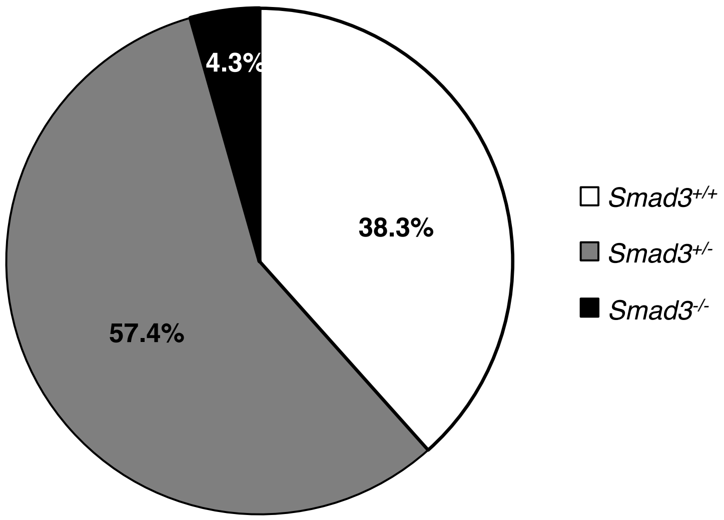

Supplementary Figure 27 Low birth rate of *Smad3*$^{-/-}$ mice with C57BL/6 background. Percentages of born genotypes of *Smad3* mice (*Smad3*$^{+/+,\ +/-,\ -/-}$) are shown (*n* = 501).

Supplementary Tables

Supplementary Table 1 Primer sequences for quantitative RT-PCR

Gene	Sense primer	Antisense primer
Gapdh	TGGTGAAGGTCGGTGTGAAC	CCATGTAGTTGAGGTCAATGAAGG
Il17a	CTCCAGAAGGCCCTCAGACTAC	AGCTTTCCCTCCGCATTGACACAG
Rorc	CCGCTGAGAGGGCTTCAC	TGCAGGAGTAGGCCACATTACA
Batf	CCCAAAGCAGCGAGATGT	GGTTCTAATCTTCCGGGCTC
Il6	GAGGATACCACTCCCAACAGACC	AAGTGCATCATCGTTGTTCATACA
Il6ra	GCACGTGGTCCAGGTCCGTG	GGGGCGAGGACACTCGTTGC
Il23r	GTCCACCAAACTTCCCAAGA	ACATGATGGCCAAGAAGACC
Il21	CGCCTCCTGATTAGACTTCG	TGTTTCTTTCCTCCCCTCCT
Il21r	ATGGCTATCCAGCCATGAAC	GGGTCCTTCATCAGTCTCCA
Il2	CCCACTTCAAGCTCCACTTC	ATCCTGGGGAGTTTCAGGTT
Il2ra	AGAACACCACCGATTTCTGG	AGCTGGCCACTGCTACCTTA
Tbet	GCCAGGGAACCGCTTATATG	GACGATCATCTGGGTCACATTGT
Eomes	TGAATGAACCTTCCAAGACTCAGA	GGCTTGAGGCAAAGTGTTGACA
Pias3	GGACGTGTCCTGTGTGTGAC	CTCTGATGCCTCCTTCTTGG

Supplementary Table 2 Primer sequences for the proximal promoter regions

**the *Rorc* promoter**	**Sense primer**	**Antisense primer**
-2.0 kb	AAAGCTAGCTTGGTGTTCATCTCTGTGGT	AAAGATATCGACTGAGAACTTGGCTCCCT

**the *Il17a* promoter**	**Sense primer**	**Antisense primer**
-2.0 kb	AAACTCGAGTAACAACAACAACAACAAAAG	AAAAAGCTTGTTTGCGCGTCCTGATCAGCTG

Supplementary Table 3 Primer sequences for ChIP

the *Rorc* promoter	Sense primer	Antisense primer
-1499 to -1304	AGTCTCAACAATGGGGTCGT	CGTGTGAGTGTGCATGTCTG
-1331 to -1170	ATCCTCCACAGACATGCACA	GTTCTTAGCCCCAGGGAGAC
-1071 to -853	CAACGGTGGAGAATGGAATG	TTCCTGCTTACCCAACAACC
-230 to -44	AATCACTCGTGCCTGTAGGG	GAGGGGACTCAGGGAGAGAG

The *Il17a* promoter	Sense primer	Antisense primer
-1958 to -1795	CCCTATGCAGTTGGTACAAAGA	TCTCTCCAGCTCCATGGATTA
-1847 to -1731	GCCACATACCAAAGAGACAAATGA	TGGTTTCTGGGAATTGAACTCA
-279 to -112	GCAGCAGCTTCAGATATGTCC	TGAGGTCAGCACAGAACCAC
-184 to -10	AACTTCTGCCCTTCCCATCT	GCTCCTTTCTCTCTTTTTATACGG
+4 to +59	CACCTCACACGAGGCACAAG	ATGTTTGCGCGTCCTGATC
